# Pathogenic Leptospires Limit Dendritic Cell Activation Through Avoidance of TLR4 and TRIF Signaling

**DOI:** 10.3389/fimmu.2022.911778

**Published:** 2022-06-22

**Authors:** Julie Cagliero, Frédérique Vernel-Pauillac, Gerald Murray, Ben Adler, Mariko Matsui, Catherine Werts

**Affiliations:** ^1^ Institut Pasteur de Nouvelle-Calédonie, member of the Pasteur Network, Immunity and Inflammation Group (GIMIN), Noumea, New Caledonia; ^2^ Institut Pasteur, Université de Paris, CNRS UMR6047, INSERM U1306, Unité de Biologie et Génétique de la Paroi bactérienne, F-75015 Paris, France; ^3^ Institut Pasteur de Nouvelle-Calédonie, member of the Pasteur Network, Leptospirosis Research and Expertise Unit, Noumea, New Caledonia; ^4^ Department of Microbiology, Biomedicine Discovery Institute, Monash University, Melbourne, Australia

**Keywords:** dendritic cells, *Leptospira*, toll-like receptor 4, TRIF, immune evasion, CD40, DC-SIGN, MHC-II

## Abstract

*Leptospira interrogans* is a bacterial species responsible for leptospirosis, a neglected worldwide zoonosis. Mice and rats are resistant and can become asymptomatic carriers, whereas humans and some other mammals may develop severe forms of leptospirosis. Uncommon among spirochetes, leptospires contain lipopolysaccharide (LPS) in their outer membrane. LPS is highly immunogenic and forms the basis for a large number of serovars. Vaccination with inactivated leptospires elicits a protective immunity, restricted to serovars with related LPS. This protection that lasts in mice, is not long lasting in humans and requires annual boosts. Leptospires are stealth pathogens that evade the complement system and some pattern recognition receptors from the Toll-like (TLR) and Nod-Like families, therefore limiting antibacterial defense. In macrophages, leptospires totally escape recognition by human TLR4, and escape the TRIF arm of the mouse TLR4 pathway. However, very little is known about the recognition and processing of leptospires by dendritic cells (DCs), although they are crucial cells linking innate and adaptive immunity. Here we tested the activation of primary DCs derived from human monocytes (MO-DCs) and mouse bone marrow (BM-DCs) 24h after stimulation with saprophytic or different pathogenic virulent or avirulent *L. interrogans*. We measured by flow cytometry the expression of DC-SIGN, a lectin involved in T-cell activation, co-stimulation molecules and MHC-II markers, and pro- and anti-inflammatory cytokines by ELISA. We found that exposure to leptospires, live or heat-killed, activated dendritic cells. However, pathogenic *L. interrogans*, especially from the Icterohaemorraghiae Verdun strain, triggered less marker upregulation and less cytokine production than the saprophytic *Leptospira biflexa*. In addition, we showed a better activation with avirulent leptospires, when compared to the virulent parental strains in murine BM-DCs. We did not observe this difference in human MO-DCs, suggesting a role for TLR4 in DC stimulation. Accordingly, using BM-DCs from transgenic deficient mice, we showed that virulent Icterohaemorraghiae and Manilae serovars dampened DC activation, at least partly, through the TLR4 and TRIF pathways. This work shows a novel bacterial immune evasion mechanism to limit DC activation and further illustrates the role of the leptospiral LPS as a virulence factor.

## Introduction

Leptospirosis is a life-threatening zoonosis of global distribution, responsible for 1 million cases, and almost 60 000 human deaths, every year (1). This disease mostly affects vulnerable populations and livestock, causing a largely underestimated public health issue as well as a considerable economic burden (2, 3).

Leptospirosis is caused by pathogenic spirochete bacteria from the genus *Leptospira* that can survive for weeks in the environment in soil and water (4). Leptospires can infect vertebrates through abraded skin or mucosa. Humans, along with other susceptible mammals, display symptoms ranging from mild, with flu-like manifestations, to hemorrhages, multi-organ failure and a dramatically increased fatality rate (5). In contrast, reservoir animals, including rats and mice, are asymptomatic renal carriers of the bacteria and shed leptospires in their urine, thus contaminating the environment. Other animals such as cattle may present with infection of the upper genital tract, sometimes asymptomatic without renal colonization or potentially leading to abortions (6, 7). Of note, the very high number of circulating serovars of pathogenic leptospires constitutes a hurdle in the development of universal vaccines (8). Vaccination with inactivated *Leptospira* strains leads to a serovar-specific, T-cell independent, humoral immunity, directed mostly against the lipopolysaccharide (LPS), that confers short-lasting protection (9). However, a recent study suggests that immunization or infection with live or inactivated leptospires confers a potent specific immunity in mice, since mice challenged 6 months post-infection survived a homologous lethal challenge (10).

Upon infection with a microorganism, the first line of defense relies on the innate immune response, which is triggered by the recognition of microbial-associated molecular patterns (MAMPs) by pattern recognition receptors (PRRs). Besides activation in the blood of the complement system leading to microbe destruction, MAMPs interaction with cellular PRRs activates signaling cascades that lead to the production of inflammatory mediators as well as the recruitment and activation of innate immune cells, especially neutrophils, macrophages and dendritic cells (DCs).

Among innate immune cells, DCs play a pivotal role in the initiation and shaping of the adaptive immune response. DCs express myeloid markers such as CD11c and a large number of co-stimulation molecules and PRRs at their surface, including C-type lectins such as the DC-Specific intercellular adhesion molecule-3-Grabbing Nonintegrin (DC-SIGN or CD 209), binding carbohydrates. Upon PRR/MAMP recognition, DCs induce pathogen endocytosis and processing through the endolysosomal pathway, which leads to MHC class II-mediated antigen presentation to CD4+ T cells. In addition, DCs and T cells produce cytokines that trigger the adaptive immune response, leading to specific antibody production by B cells and cellular responses.

Both acute symptomatic disease and asymptomatic renal colonization support the notion that leptospires can evade host immune defenses. Growing evidence suggests that pathogenic leptospires, as to other spirochetes, have evolved various strategies to establish infection successfully. First, pathogenic, but not saprophytic, leptospires are notably able to evade complement (11). Further, these pathogenic bacteria largely escape from phagocyte recognition and processing unless opsonized with specific antibodies (12). Investigating how these pathogens escape from immune defenses is crucial, as it is likely to be the key to understanding the differential susceptibility between hosts to leptospirosis as well as the ability of leptospires to colonize renal tubules of reservoir animals (8). Due to particular features of their cell wall, such as an atypical LPS and presence of numerous lipoproteins, it has been shown that leptospires escape the innate immune recognition from several Toll-like (TLR) and NOD-like (NLR) receptors (8). Interestingly, some of these escape mechanisms are host-specific. For example, leptospires escape the TLR4 response in human cells, but are recognized by the mouse TLR4 although they avoid the endocytic TLR4/TRIF pathway, due to the O-antigen part of their LPS (13, 14). Moreover, once they are inactivated, leading to the endoflagella disruption and exposure, leptospires are recognized by the human and bovine TLR5 but barely by mouse TLR5 (15). In addition to the host specificity of PRR recognition of leptospires, species specificities have also been noticed. Indeed, it was recently highlighted that the long-term humoral response in mice after infection with pathogenic *L. interrogans* is not, qualitatively and quantitatively, equivalent between different serovars of *L. interrogans*. These data suggest that different serovars induce differential host immune responses, according to the particular pathophysiology triggered by the different serovars of leptospires (10).

To date, knowledge on the recognition and processing of leptospires by DCs remains extremely limited. However, DCs are among the first immune cells proposed to encounter leptospires in the course of an infection, and Langerhans cells are particularly potent antigen presenting DCs in the skin and mucosa. In 2008, Gaudart and colleagues showed that DC-SIGN could bind to both virulent and avirulent strains of leptospires *in vitro*, with levels of affinity varying between serovars (16). Further data suggested that leptospires were able to induce maturation of human MO-DCs, although these processes seem to show differences depending on the serovar. Since then, DC activation in the presence of leptospires has not been explored further. Therefore, we hypothesized that evasion or incomplete processing of leptospires by DCs could account for differences in immune response and immune memory between humans and mice.

Hence, in this work, we used DCs, derived from humans and mouse primary cells, to measure and compare their activation state, through the quantification of cell surface markers and cytokine production, upon exposure to different serovars of *L. interrogans.* Results were compared to the maturation of DCs exposed to saprophytic leptospires or avirulent strains. Our results suggest that, although exposure to *Leptospira* activates DCs, this activation seems to be limited in the presence of pathogenic leptospires, when compared to saprophytic or avirulent leptospires.

## Materials and Methods

### Ethical Considerations

New Caledonian local ethic committee authorization was obtained for the use of human leucocytes from healthy platelet- donors after cytapheresis at the Territorial Hospital Center in New Caledonia. All methods and all experimental protocols were carried out in accordance with relevant human ethical guidelines and regulations of the Institut Pasteur of New Caledonia (IPNC). The donors consented that their blood would be used for research.

Animal studies were conducted according to the guidelines of the Animal Care and Use Committees of the Institut Pasteur and followed European Recommendation Directive 2010/63/EU.

Experiments with C57BL/6J mice were carried out under the protocol number HA0036, approved by the Institut Pasteur ethics committee (CETEA 89) (Paris, France).

All experiments have been performed on human and animal cells of both sexes.

### Animals

Ten to twenty-week-old Outbred OF1 mice (*Mus musculus*) and golden Syrian hamsters (*Mesocricetus auratus*), initially obtained from Charles River Laboratories, were bred in the animal facility at IPNC. Eight to twelve-week-old female and male C57BL/6J (B6) mice were purchased from Janvier Labs (Le Genest Saint Isle, France) and maintained in accordance with regulatory requirements until use. Age and gender matched TLR4 deficient mice (TLR4^-/-^) or mutant TRIF/*Lps2* mice, both in the B6 background, were bred by the Institut Pasteur animal facility, and were described previously (17).

### Leptospira Strains

All strains of *Leptospira* were grown in Ellinghausen McCullough Johnson Harris (EMJH) medium and incubated at 30°C without agitation in aerobic conditions. Cultures were passaged weekly (twice a week for *L. biflexa*) by a 1/200 dilution to obtain late log phase cultures at the time of experiments. At IPNC, L495 and Verdun cultures were passaged in hamsters for virulence maintenance and used up to a maximum of ten *in vitro* passages. Two different serovars of the pathogenic species *L*. *interrogans* (including wild-type strains and mutants) and one serovar of saprophytic *L*. *biflexa* were used (**Table 1**).

**Table 1:** *Leptospira* strains used for DCs challenge at the Institut Pasteur in Paris (IPP) and at the Institut Pasteur of New Caledonia (IPNC).

*Leptospira* strains - IPP					
	serovar	strain	Virulence	Designation	Passage into animals
*L. interrogans*	Icterohaemorrhagiae	Verdun (Clone 3) (10)	Pathogenic Low passage	Verdun vir	No
*L. interrogans*	Icterohaemorrhagiae	Verdun (Clone 3 p104) (10)	Avirulent	Verdun avir	No
*L. interrogans*	Manilae	L495	Pathogenic low passage	L495	No
*L. interrogans*	Manilae	M895 mutant of L495 (18–20)	Avirulent	M895	No
*L. biflexa*	Patoc	Patoc1	Saprophytic	Patoc	No
***Leptospira* strains - IPNC**					
	**serovar**	**strain**	**Virulence**	**Designation**	**Passage into animals**
*L. interrogans*	Icterohaemorrhagiae	Verdun CNR	Pathogenic Low passage	Verdun vir	Yes
*L. interrogans*	Icterohaemorrhagiae	Verdun	Avirulent High passaged	Verdun avir	No
*L. interrogans*	Manilae	L495	Pathogenic low passage	L495	Yes
*L. biflexa*	Patoc	Patoc1	Saprophytic	Patoc	No

Immediately before cell stimulation, bacteria were centrifuged at 3,125 g for 20 minutes at room temperature. Bacteria were then resuspended in PBS, counted using a Neubauer or Petroff-Hauser chamber, and density was adjusted in cell culture media (RPMI or IMDM) without antibiotics to the required multiplicity of infection (MOI). For the heat-killed (HK) conditions, bacterial suspensions were heated at 56°C for 30 minutes.

### Cell Culture

The bone-marrow derived DCs (BM-DCs) and monocyte derived DCs (MO-DCs) in primary cultures were maintained at 37°C in a humidified 5% CO2/95% air atmosphere in culture media described in **Table 2**.

**Table 2:** Cell culture media used for DCs differentiation and activation at the Institut Pasteur in Paris (IPP) and Institut Pasteur of New Caledonia (IPNC).

	BM-DCs derived from C57BL/6 mice (IPP)	BM-DCs derived from OF1 mice (IPNC)	Human MO-DCs (IPNC)
Medium	IMDM (Gibco #12440053)		RPMI 1640 (Sigma #R8758)
Supplements	1X NEAA (Gibco #11140-035) 1 mM Sodium Pyruvate (Gibco #11360-070) 10 mM HEPES (Gibco #15630-056 or Sigma #H0887)		
	Penicillin 100 U/mL, Streptomycin 100 µg/mL 10% FBS (Gibco #1082-147)	Penicillin 100 U/mL, Streptomycin 100 µg/mL, Amphotericin B 250 ng/mL (Sigma #5955) 10% FBS (Dutscher #S181-B500)	
Differentiation factors	20 ng/mL rmGM-CSF (PeproTech # 315-03)	20 ng/mL rmGM-CSF (Miltenyi # 130-095-739)	800 IU/mL rhGM-CSF (Miltenyi # 130-093-866) 250 IU/mL rhIL-4 (Miltenyi # 130-093-922)
Designation	**IMDMc**		**RPMIc**

### Bone Marrow Progenitor Cells Isolation and Differentiation

Bone marrow was harvested by flushing the femurs of mice with a 2.5 ml syringe filled with IMDM, as described previously (15). Marrow was passed through a 70 µm cell strainer and recovered bone marrow cells were centrifuged at room temperature for 7 minutes at 350 g. After red blood cell lysis using Red Blood Cell lysis buffer (Sigma-Aldrich) and cell counting using Trypan blue, 5 x 10^6^ viable bone marrow cells were seeded in 10 mL of IMDMc in a non-treated 10 cm Petri Dish and cultured for 7 days. Half of the medium was changed every 2-3 days (IPNC). Every 2-3 days, 3 to 5 mL of IMDMc were added to the culture (IPP).

### Monocyte Isolation From PBMCs

PBMCs were isolated from healthy donor buffy coats using Hispaque-1077 (Sigma-Aldrich) following the manufacturer’s protocol. Red blood cells (RBC) of single cell suspensions were lysed using RBC Lysis Buffer (Sigma-Aldrich). Monocytes were isolated from PBMCs by negative isolation using the Dynabeads Untouched Human Monocytes Kit (Thermofischer scientific) according to the manufacturer’s instructions. Briefly, an antibody mix directed towards non-monocytes (T cells, B cells, NK cells, dendritic cells, granulocytes, and erythrocytes) was added to PBMCs in suspension and allowed to bind to cells. PBMCs were then washed, and magnetic Dynabeads were added and allowed to bind to antibody-labeled cells. Bead-bound cells were separated from the cell suspension using a magnet, and remaining untouched monocytes were quickly counted using Trypan blue and seeded in 6 well plates at the density of 1 x 10^6^ cells/mL of RPMIc with rhGM-CSF and rhIL-4 and cultured for 7 days. Half of the medium was renewed every 2-3 days.

### Stimulation

On day 7, non-adherent and loosely adherent human or mouse cells were gently harvested, centrifuged for 7 minutes at 350 g, and resuspended in fresh medium without antibiotics. Cells were seeded in 24 or 6 well plates at a density of 8 x 10^5^ – 10^6^ viable cells/mL per well and left for a 24h, after which human or mouse cells were used as the starting immature material in the MO-DCs and BM-DCs experiments, respectively.

On day 8, BM-DCs or MO-DCs were stimulated with leptospires at a MOI of 100, 50 or 10 live bacteria per cell, or at a MOI of 100 or 10 heat-killed (HK) leptospires/cell, or with *Escherichia coli* lipopolysaccharide (LPS-EB from *E. coli* O111:B4, InvivoGen, 1 µg/mL) in 100µL of complete fresh medium. Cells were stimulated for 24h.

On day 9, supernatants were harvested and immediately stored at -20°C for subsequent cytokine quantification. BM-DCs or MO-DCs were incubated in Versene buffer or Cell Dissociation Buffer (Gibco), non-enzymatic cell dissociation reagents, for 15-30 minutes at 37°C. Cells were then flushed, harvested, centrifuged for 7 minutes at 350 g and immunostained for subsequent flow cytometry analysis.

### Flow Cytometry

Cell surface staining was performed in ice-cold PBS containing 2% FBS and 2 mM EDTA (cytometry buffer) using appropriate fluorochrome-conjugated monoclonal antibodies and corresponding isotypes, all purchased from BD Biosciences (**Table 3**). A total of 1.5 to 2×10^5^ cells per experimental condition were resuspended in cytometry buffer containing 2 µg/mL of FcBlock (anti CD16/CD32) for 20 min at 4°C, then stained with 1 µg/mL of antibody or control isotypes for 30 min on ice in the dark. Next, cells were washed with cytometry buffer, and fixed in BD cell fix buffer (BD Biosciences) or in 4% paraformaldehyde solution before acquisition through a BD FACSCanto™ II Cell Analyzer (IPNC) or a CytoFLEX flow cytometer (Beckman Coulter). For cell viability analysis, cells were incubated with eBioscience™ Fixable Viability Dye eFluor 780 for 5 min on ice, washed in cytometry buffer before fixation. Positive eFluor dead cells were excluded from the analysis. At least 30,000 events were acquired for the phenotyping. Compensations were performed using UltraComp eBeads (Thermofisher Scientific) stained positively with each of the antibodies used in the experiment. Baseline photomultiplier tube voltages of the instrument were set using unstained cells, showing the background fluorescence of the experimental samples. Data were analyzed using FlowJo v10 analysis software package.

**Table 3:** Antibodies used for flow cytometry.

Fluorochrome	Antibody	BD Biosciences Cat #	
		Mouse	Human
FITC	CD11b Isotype	553310 556923	- -
FITC	DC-SIGN Isotype	- -	551264 555748
PE-Cy7	CD11c Isotype	558079 557798	561356 557872
PerCP-Cy5	MHC-II/HLA-DR Isotype	107625 550764	560652 550927
BV421	F4/80 Isotype	8565411 562602	- -
BV421	CD86 Isotype	- -	562432 562438
APC	CD40 Isotype	558695 553932	555591 555751
APC	DC-SIGN Isotype	564928 553932	- -
PE-CF594	CD83 Isotype	- -	562631 562292
PE	CD80 Isotype	553769 550085	- -

### Cytokine Analysis

The amount of TNF-α, IL-6, IL-12p40 and IL-10 were quantified from adapted dilutions of cell-free culture supernatants using mouse-specific or human-specific DuoSet ELISA kits (R&D) according to the manufacturers’ instructions. References of commercial ELISA kits are listed in **Table 4**.

**Table 4:** ELISA kits used for cytokine quantification in cell supernatants.

Cytokine	R&D Cat #	
	Mouse	Human
TNF-α	DY410	DY210
IL-6	DY406	DY206
IL-12/IL-23 p40	DY2398	DY1270
IL-10	DY417	DY217B

### Statistical Analysis

Data were depicted in figures as the mean along with standard deviation. Level of significance for comparison between conditions was determined by nonparametric Kruskal-Wallis comparison test followed by post-hoc Fisher’s Least Significant Difference (LSD) test. All statistical analyses were performed with R software using the lmtest package from the CRAN project. This method uses the Piepho (2004) algorithm (as implemented in the multcompView package) to generate a compact letter display of all pairwise comparisons of estimated marginal means (21). Statistics indicated with lower-case letters allow to compare, for each marker or cytokine, all conditions of stimulation within live (upper panel) and eventually heat-killed bacteria (lower panel). Conditions that share the same lower-case letters are not significantly different (p > 0.05). In contrast, conditions that do not share same letter(s) are significantly different.

## Results

### Activation Profiles of Dendritic Cells Derived From Bone Marrow Cells of Mice and Human Monocytes

Dendritic cells were derived from bone marrow cells (BM-DCs) of male and female C57BL/6 (B6) and OF1 mice, two mouse lines of different genetic backgrounds, and from human circulating monocytes purified from healthy blood donors of both sexes (MO-DCs). Those immature cells were then stimulated with LPS from *E. coli* for 24h to validate their ability to be activated into mature DCs, to express more cell surface activation markers, and to produce inflammatory cytokines. The expression of lineage (CD11b and CD11c for BM-DCs and CD11c and HLA-DR for MO-DCs) and activation markers of DCs (DC-SIGN, CD40, CD80 and MHC-class II (MHC-II) for BM-DCs; DC-SIGN, CD40, CD83, CD86 and HLA-DR for MO-DCs) were quantified by flow cytometry, compared to isotype controls. The results are presented in **Supplementary Figure 1**. Since histograms of mouse DCs showed a non-gaussian fluorescence spectrum upon LPS stimulation (**Supplementary Figure 1Ab**), we chose not to quantify Mean or Median Fluorescence Intensity (MFI). Consequently, all flow cytometry results for marker expression are expressed as a percentage of positive cells in the CD11b^+^/CD11c^+^ and CD11c^+^/HLA-DR^+^ populations.

The gated mouse CD11b^+^/CD11c^+^ BM-DCs population represented around 63% and 42% of the unstimulated cells in B6 and OF1 mice, respectively (**Supplementary Figures 1Ac, Bc**). In human cells, the gated CD11c^+^/HLA-DR^+^ population, considered as our MO-DC model, represented 79% of unstimulated cells (**Supplementary Figure 1Cc**). Equivalent activation markers between mouse and human models were expressed similarly in unstimulated cells, with very low expression of DC-SIGN and CD40, that were greatly upregulated upon LPS stimulation in BM-DCs, but not in MO-DCs, and high expression of CD80 in mouse DCs and CD86 in MO-DCs that were upregulated by LPS, in a lesser extent (**Supplementary Figure 1A**).

As described (22), we observed in B6 BM-DCs a slight downregulation of CD11c upon LPS stimulation (**Supplementary Figure 1A**). Of note, stimulation for 24h of with LPS or infection with leptospires did not affect the viability of MO-DCs and total derived cells from OF1 bone marrow (**Supplementary Figure 1D**).

### Leptospires Differently Upregulate Activation Markers on Murine B6 BM-DCs

**Figure 1:**
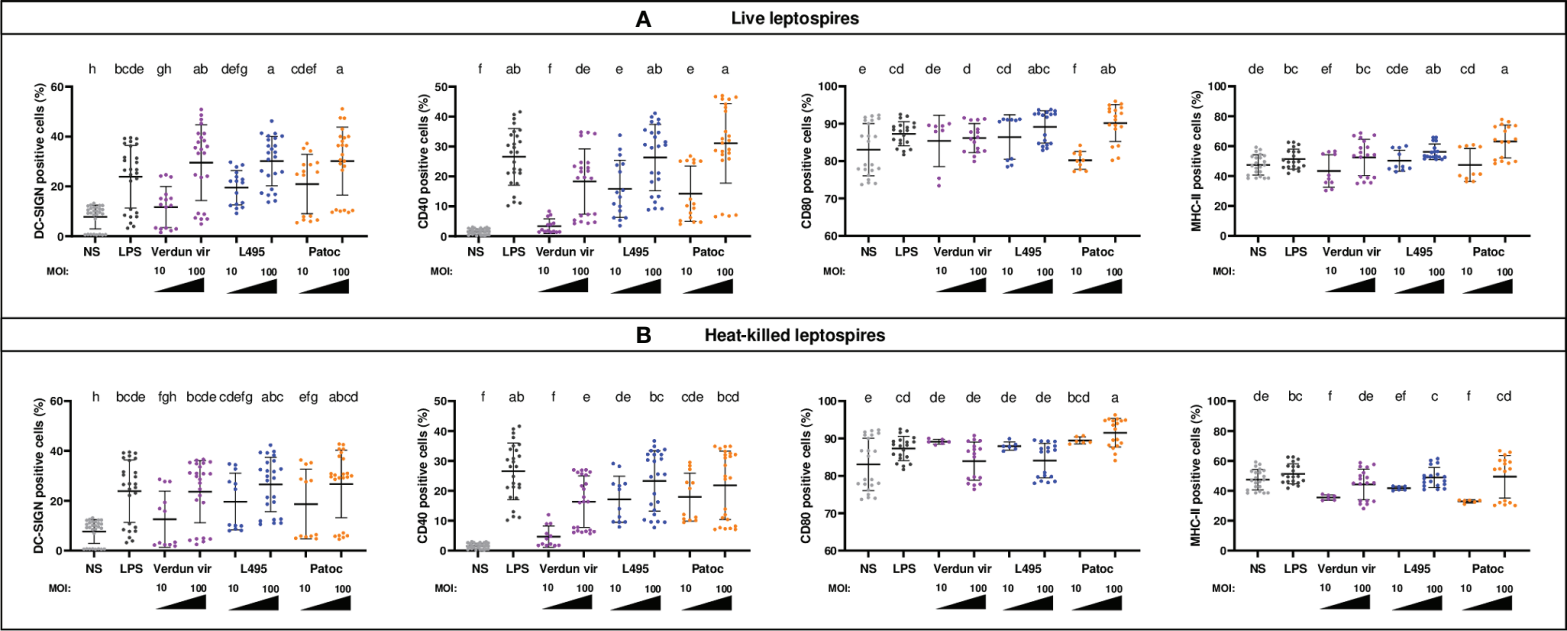
BM-DCs from C57BL/6 mice exposed to live and heat-killed leptospires express more cell surface markers. Day 8 BM-DCs from C57BL/6 mice were exposed for 24h to either live **(A)** or heat-killed **(B)** Icterohaemorrhagiae Verdun vir, Manilae L495 and Patoc leptospires at a Multiplicity of Infection (MOI) of 10 or 100 bacteria per cell. *E*. *coli* LPS (1 µg/mL) was used as a positive control for DCs stimulation. After 24h, cells were immunostained for the analysis of the expression of DC-SIGN, CD40, CD80 and MHC-II markers by flow cytometry and data were compared to results with non-stimulated cells (NS). Data are represented in dot plot diagrams showing the percentage of BM-DCs positive for DC-SIGN, CD40, CD80 and MHC-II staining. Each dot represents one out of four replicates of stimulation from five independent experiments with n=6 mice. Mean ± standard deviation (SD) is also represented (black lines). Statistics indicated above graphs with lower-case letters allow for comparing and ranking (from a, the most upregulated to h, the less upregulated) for each marker, all conditions within live (upper panel) and heat-killed (lower panel). Conditions that share the same lower-case letters are not significantly different (p > 0.05). In contrast, conditions that do not share same letter(s) are significantly different.

To evaluate the ability of leptospires to activate dendritic cells, the derived DCs were exposed for 24h to leptospires, live or heat-inactivated, and at different MOI. Different species of leptospires were used. *L. interrogans* is a pathogenic species responsible for the most severe forms of leptospirosis, while *L. biflexa* is saprophytic and does not cause disease. We chose to study the strain *L. biflexa* Patoc I (Patoc) and two serovars of *L. interrogans*, Manilae strain L495 (L495) and Icterohaemorrhagiae strain Verdun (Verdun), responsible for severe human diseases in East-Asia and Europe, respectively. In BM-DCs from B6 mice, exposure to L495 and Patoc live leptospires upregulated in a dose-dependent manner the percentage of cells positive for DC-SIGN, CD40 and CD80 activation markers (**Figure 1A**). Percentage of MHC-II^+^ cells was also increased after exposure to leptospires at MOI 100, but not at MOI 10 (**Figure 1A**). The activation of BM-DCs was significant, since at MOI 100, the magnitude was comparable to that observed after exposure to *E. coli* LPS used as a control. Exposure to Verdun induced a lower, but significant, activation of BM-DCs. Indeed, no change in the expression of activation markers was observed with Verdun at MOI 10, and the increase of CD40^+^, CD80^+^ and MHC-II^+^ cells at MOI 100 were comparable to the activation obtained after exposure to L495 at MOI 10 (**Figure 1A**). In contrast, DC-SIGN was equally upregulated by all leptospires at MOI 100.

Leptospiral vaccines are often made of whole inactivated leptospires that are not infectious and may present some denatured MAMPs. To test whether the DC activation would be different with inactivated bacteria, we performed the experiments in parallel with heat-killed (HK) leptospires (**Figure 1B**). Compared to live strains, no major differences were observed with pathogenic HK strains for DC-SIGN and CD40 markers (**Figures 1A, B**). In contrast, loss of upregulation or even a decrease in MHC-II^+^ and CD80^+^ cells were observed in the presence of HK pathogenic leptospires at MOI 100 (**Figures 1A, B**). However, as compared to pathogenic strains, the saprophytic strain behaved differently, since upon stimulation with HK Patoc at MOI 100, we observed a decrease in CD40^+^ cells, and a higher expression of CD80 compared to live bacteria (**Figure 1**).

### Leptospires Trigger Cytokine Secretion by Mouse B6 BM-DCs

**Figure 2:**
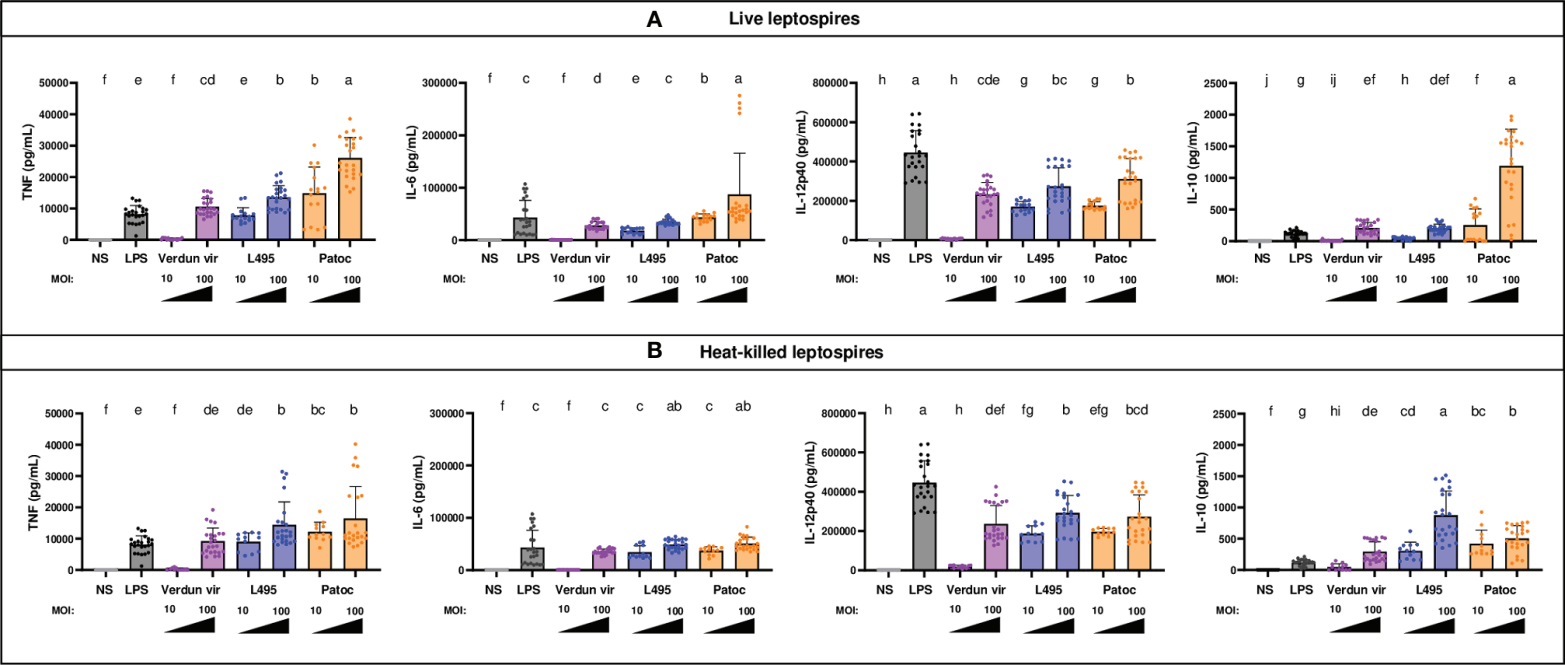
BM-DCs from C57BL/6 mice exposed to live and heat-killed leptospires produce more pro- and anti-inflammatory cytokines. Day 8 BM-DCs from C57BL/6 mice were exposed to either live **(A)** or heat-killed **(B)** Icterohaemorrhagiae Verdun vir, Manilae L495 and Patoc leptospires at a Multiplicity of Infection (MOI) of 10 or 100 bacteria per cell for 24h. *E*. *coli* LPS (1 µg/mL) was used as a positive control for DCs stimulation. After 24h, cytokine concentrations were quantified in cell supernatants by ELISA assays and data were compared to results with non-stimulated cells (NS). Data are represented in histograms with dot plot showing the concentration of pro-inflammatory TNF, IL-6 and IL-12p40 and anti-inflammatory IL-10 in cell supernatants. Each dot represents one out of four replicates of stimulation from five independent experiments with n=6 mice. Mean ± SD is also represented. Statistics indicated with lower-case letters allow for comparing, for each cytokine all conditions within live (upper panel) and heat-killed (lower panel). Conditions that share the same lower-case letters are not significantly different (p > 0.05). In contrast, conditions that do not share same letter(s) are significantly different.

A dose dependent production of pro-inflammatory (TNF, IL-6 and IL-12p40) and anti-inflammatory (IL-10) cytokines in BM-DCs supernatants was observed after infection with all leptospires (**Figure 2A**). Stimulation with saprophytic Patoc triggered more cytokine production than the pathogenic strains. This difference was particularly marked for the anti-inflammatory IL-10, since BM-DCs exposed to Patoc at MOI 10 produced the same amount of IL-10 as cells exposed to pathogenic leptospires at MOI 100 (**Figure 2A**). Interestingly, exposure to Verdun triggered less production of cytokines, since no significant cytokine release was quantified at MOI 10, in contrast to the other strains. These results are consistent with the lower increase of cells positive for activation markers (**Figure 1**).

Of note, the IL-12p40 production was relatively equivalent between all strains at MOI 100, including Patoc, but lower than that seen with LPS, contrary to the production of the other cytokines that were produced in equivalent or higher amounts compared with LPS (**Figure 2A**). Interestingly, although the cytokine secretion was equivalent between live and HK strains for TNF, IL-6 and IL-12p40, the IL-10 production at MOI 100 was higher with HK Manilae and lowered with HK Patoc compared to live bacteria (**Figures 2A, B**).

### Leptospires Activate Mouse OF1 BM-DCs

To study the DC response in another mouse primary cell model, expression of DC-SIGN, CD80 and MHC-II markers and cytokine secretion were studied in OF1 BM-DCs upon stimulation with live and HK leptospires. All leptospires stimulated in a dose dependent manner the expression of all the markers and cytokines (**Supplementary Figures 2B, C**). The comparative statistics showed that cells were less activated after exposure to Verdun leptospires. Indeed, Verdun only upregulated CD80 at MOI 100, and in contrast with L495, did not significantly upregulate DC-SIGN and MHC-II (**Supplementary Figure 2B**). The cytokine profiles also showed a dose-dependent response, with Patoc and L495 triggering more cytokines than Verdun (**Supplementary Figure 2C**). Compared to live bacteria, stimulation with HK leptospires triggered equivalent upregulation of markers and cytokine production, except for HK Verdun that triggered less DC-SIGN^+^ cells, and HK Patoc, which triggered less TNF, IL6 and IL-10 secretion (**Supplementary Figures 2B, C**).

Overall, the results were consistent between the two mouse primary cell models and suggested that i) exposure to pathogenic leptospires induced a weaker activation of DCs compared to saprophytic leptospires and ii) the Icterohaemorrhagiae Verdun strain seemed to induce a lower response than the Manilae L495.

### Better Activation of Mouse OF1 BM-DCs by the Avirulent Verdun Derivative Compared to the Virulent Strain

**Figure 3:**
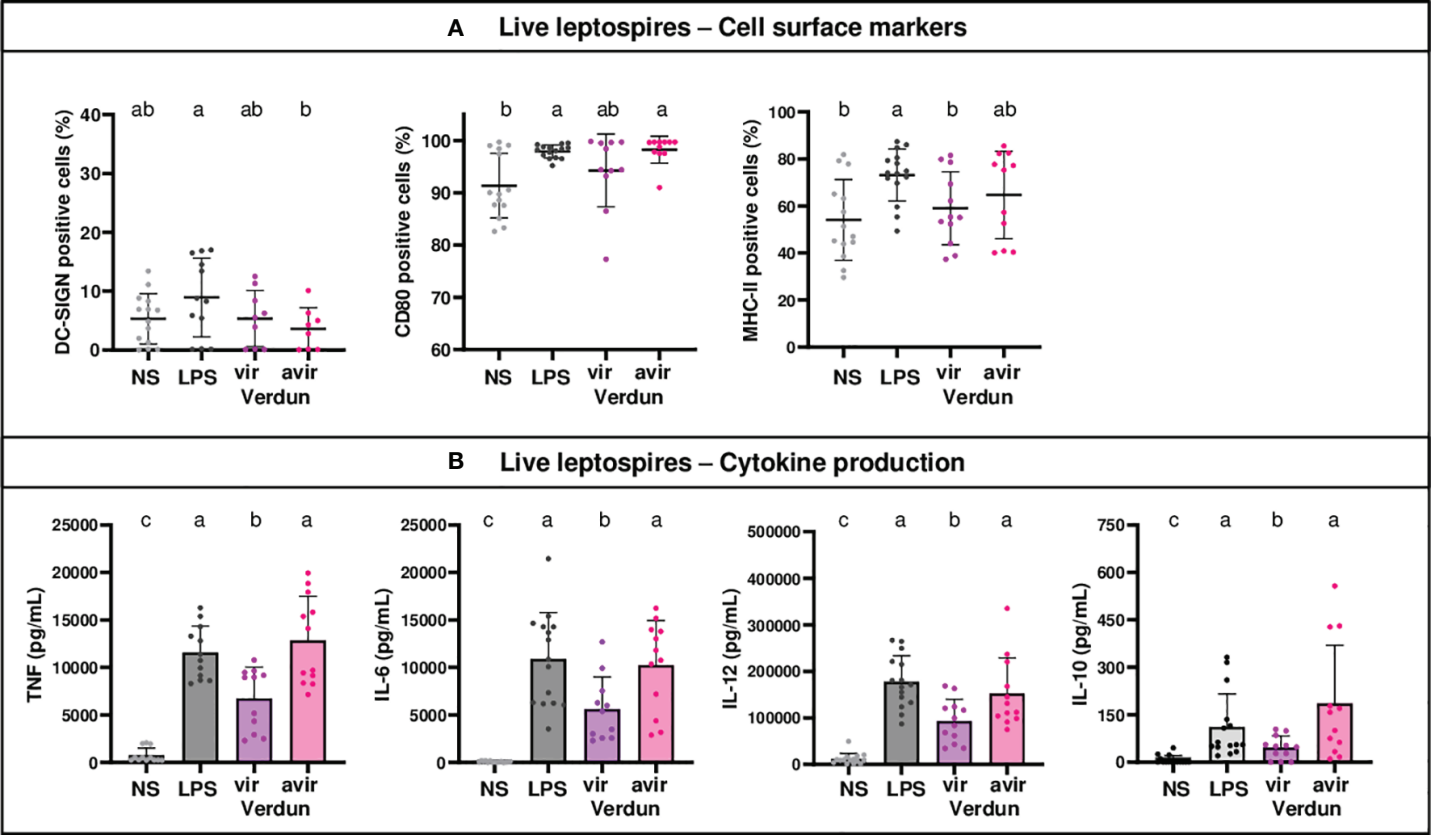
Level of activation of OF1 BM-DCs after exposure to avirulent leptospires from the Verdun strain is higher than after exposure to virulent Verdun leptospires. Day 8 BM-DCs from OF1 mice were stimulated with live virulent and avirulent leptospires from the Icterohaemorrhagiae Verdun (Verdun vir and Verdun avir) strain at a Multiplicity of Infection (MOI) of 100 bacteria per cell. *E*. *coli* LPS (1 µg/mL) was used as a positive control for DCs stimulation. After 24h, cells were immunostained for the analysis of the expression of cell surface markers **(A)** through flow cytometry and cytokine concentrations **(B)** were also quantified in cell supernatants by ELISA assays. Data were compared to results with non-stimulated cells (NS). A: Dot plot diagrams show the percentage of BM-DCs positive for DC-SIGN, CD80 and MHC-II staining. **(B)** Histograms with dot plot show the concentration of pro-inflammatory TNF, IL-6 and IL-12p40 and anti-inflammatory IL-10 cytokines in cell supernatants. Each dot represents one out of three stimulation replicates from three independent experiments with n=3 mice. Mean ± SD is represented. For each marker or cytokines, statistics indicated with lower-case letters allow for comparing all conditions. Conditions that share the same lower-case letters are not significantly different (p > 0.05). In contrast, conditions that do not share same letter(s) are significantly different.

To better understand the phenotype obtained with the pathogenic strains, we studied the activation of BM-DCs of OF1 mice stimulated with live virulent Icterohaemorrhagiae Verdun (Verdun vir) compared to a highly passaged derivative of the virulent Verdun strain (Verdun avir) that is not pathogenic for hamsters. Although no significant differences between the two strains were observed for the cell markers (**Figure 3A**), the avirulent Verdun strain triggered significantly more cytokines than the virulent Verdun strain (**Figure 3B**). Altogether these data suggested that the pathogenic virulent Verdun strain limits the activation of BM-DCs.

### No Difference of Human MO-DCs Activation Between Verdun and Its Avirulent Derivative

**Figure 4:**
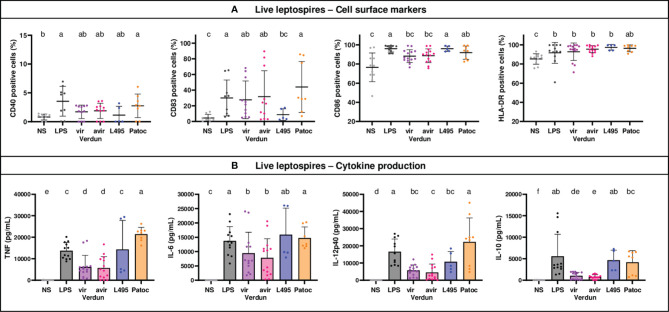
Human MO-DCs exposed to leptospires express more cell surface markers and produce more cytokines. Day 8 Human MO-DCs were stimulated with Icterohaemorrhagiae Verdun vir, Verdun avir, Manilae L495 and Patoc leptospires at a Multiplicity of Infection (MOI) of 100 bacteria per cell for 24h*. E*. *coli* LPS (1 µg/mL) was used as a positive control for DCs stimulation. After 24h, cells were immunostained for the analysis of the expression of cell surface markers **(A)** through flow cytometry and cytokine concentrations **(B)** were also quantified in cell supernatants by ELISA assays. Data were compared to results with non-stimulated cells (NS). A: Dot plot diagrams show the percentage of MO-DCs positive for DC-SIGN, CD83, CD86 staining as well as the percentage of ungated cells positive for HLA-DR staining. **(B)** Histograms with dot plot show the concentration of pro-inflammatory TNF, IL-6, and IL-12 and anti-inflammatory IL-10 in cell supernatants. Each dot represents stimulation duplicates or triplicates from independent experiments with two to five blood donors. Mean ± SD is also represented. Conditions that share the same lower-case letters are not significantly different (p > 0.05). In contrast, conditions that do not share same letter(s) are significantly different.

Using the same conditions, we also studied the activation in dendritic cells derived from human monocytes (MO-DCs) following exposure to leptospires. Since DC-SIGN was not upregulated upon LPS stimulation (**Supplementary Figure 1C**), we focused on CD40, CD83, CD86 and HLA-DR markers (**Figure 4A**). All live leptospires activated the expression of HLA-DR marker to the same magnitude as LPS, and triggered cytokine secretion (**Figure 4B**). However, leptospires differently activated the MO-DCs. The saprophytic Patoc strain was the only one to upregulate all cell markers. In addition, it triggered secretion of more pro- and anti-inflammatory cytokines than Verdun strains, and more TNF and IL12-p40 than L495. The Verdun strains did upregulate CD83 expression, although they only slightly upregulated the expression of CD86 marker, whereas it was the opposite for L495. In non stimulated MO-DCs, we measured unexpected low levels of CD40 expression, that were barely upregulated by pathogenic leptospires. The production of cytokines by MO-DCs stimulated with Verdun was reduced compared to LPS or the other leptospires strains. Interestingly, no difference in cell surface marker activation or cytokine production was observed between the Verdun virulent and avirulent derivative (**Figure 4B**). Level in cell activation was also equivalent between live and HK bacteria for the Verdun and L495 strains (**Supplementary Figure 3**).

### Differences in Mouse BM-DC Activation Between Verdun and Avirulent Derivative Rely on TLR4 Signaling

**Figure 5:**
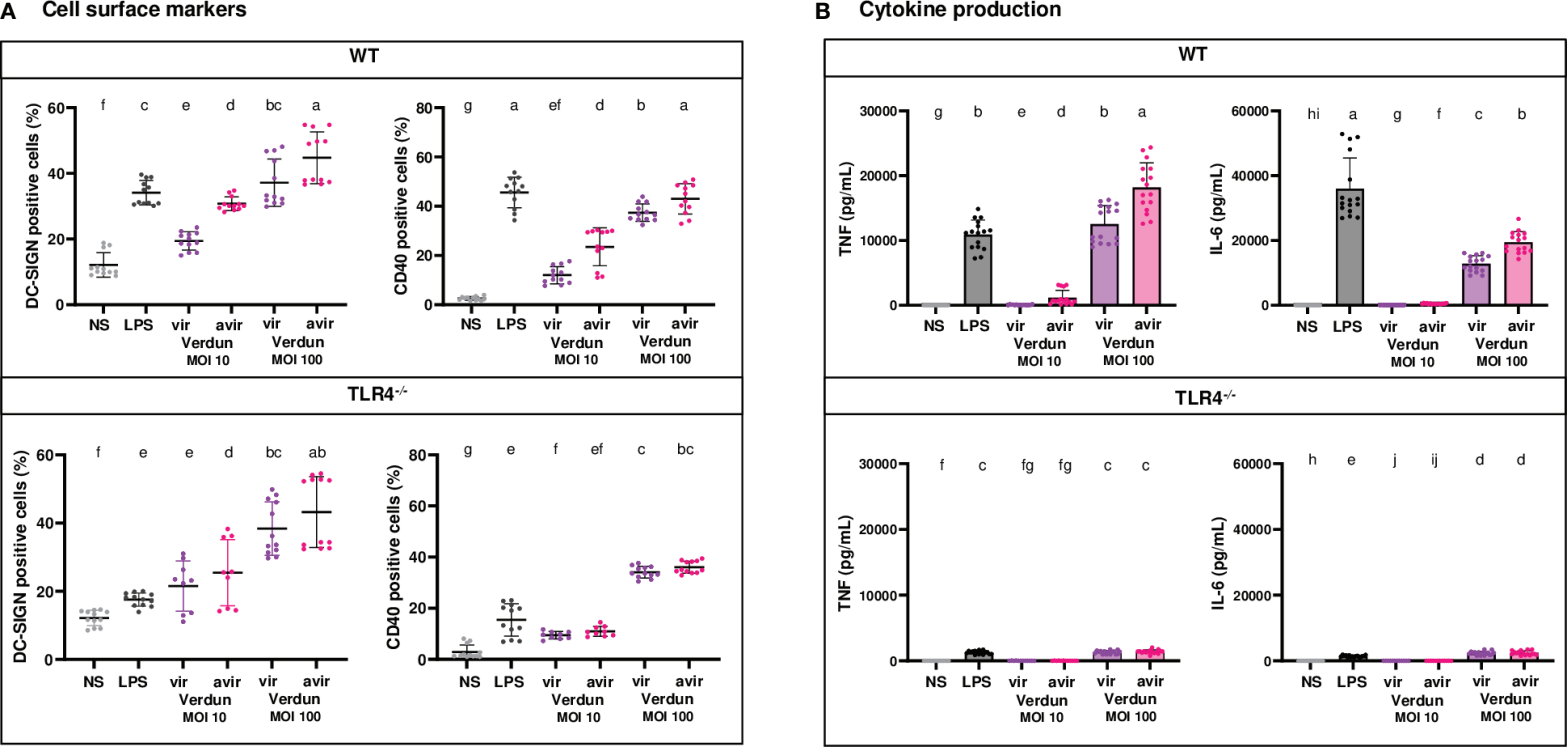
Contribution of TLR4 in the difference of BM-DCs activation upon stimulation with virulent compared to avirulent Verdun strains of leptospires. Day 8 WT and TLR4^-/-^ BM-DCs from C57BL/6 mice were stimulated with virulent and avirulent leptospires from the Icterohaemorrhagiae Verdun (Verdun vir and Verdun avir) strains at a Multiplicity of Infection (MOI) of 10 or 100 bacteria per cell. *E*. *coli* LPS (1 µg/mL) was used as a positive control for DCs stimulation. After 24h, cells were immunostained for the analysis of the expression of DC-SIGN and CD40 by flow cytometry (A, dot plot) and TNF and IL-6 concentrations were also quantified in cell supernatants by ELISA assays (B, histograms with dot plot). Data were compared to results with non-stimulated cells (NS). Each dot represents technical triplicates **(A)** or quadruplicates **(B)** from two independent experiments with n=4 mice. Mean ± SD is also represented. Statistics indicated with lower-case letters allow for comparing, for each marker and each cytokine, all conditions within WT (upper panel) and TLR4^-/-^ (lower panel). Conditions that share the same lower-case letters are not significantly different (p > 0.05). In contrast, conditions that do not share same letter(s) are significantly different.

Since the differences in DCs activation upon exposure to virulent and avirulent Verdun strains observed in the mouse model were not found in human DCs, we hypothesized that this could be due to TLR4 sensing of leptospires. Indeed, human cells are not able to recognize leptospiral LPS through TLR4 (13, 14). In addition, we recently showed in mouse macrophages that subversion of the TLR4 signaling pathway participates in the virulence of leptospires (20). To test this hypothesis, we compared activation in BM-DCs derived from WT and TLR4^-/-^ B6 mice, upon stimulation with live Verdun virulent Cl3 isolate and its avirulent derivative (Cl3 p104) obtained after 104 weekly passages *in vitro* (10) (**Figure 5** and **Supplementary Figure 4**).

First, consistent with what was observed in the OF1 BM-DCs infected with another set of Verdun strains, we also found in WT BM-DCs, exposed to the avirulent Verdun Cl3 p104 mutant, a higher dose-dependent secretion of TNF, IL-6 and IL-10 cytokines, as well as a higher CD40 and DC-SIGN upregulation compared to the parental Cl3 virulent strain (**Figure 5A** and **Supplementary Figure 4**). Interestingly, if the profile in DC-SIGN activation was equivalent in both WT and TLR4^-/-^ BM-DCs (**Figure 5A**), the difference of CD40 upregulation between the two isolates of Verdun observed in WT BM-DCs was lost in TLR4^-/-^ BM-DCs at both MOI (**Figure 5A**). In addition, although all cytokine levels in TLR4^-/-^ BM-DCs were ten times lowered compared to WT DCs, levels of TNF and IL-6 were equivalent between the virulent and avirulent Verdun strains at both MOI (**Figure 5B**) and at least for one MOI for IL-12p40 and IL-10 cytokines (**Supplementary Figure 4B**). These results suggest that the pathogenic virulent Verdun strain subverts a TLR4-dependent mechanism to dampen the BM-DCs activation, which at least for cytokine secretion, mostly relies on the TLR4 pathway.

### Some Differences of Mouse BM-DCs Activation Between Virulent and Avirulent Derivative of Leptospires Rely on TRIF Signaling

Because we previously showed in bone marrow-derived macrophages a role of the leptospiral LPS in the escape of *Leptospira* from the endosomal TLR4/TRIF pathway (20), we derived BM-DCs from mutant TRIF/*Lps2* B6 mice that do not possess a functional TRIF adaptor. We investigated their level of activation compared to WT BM-DCs upon infection at MOI 50 with two pairs of virulent and avirulent *L. interrogans*: the virulent Verdun Cl3 and avirulent Cl3 p104 strains, as well as the L495 Manilae and avirulent M895 mutant, a defined mutant obtained by random mutagenesis harboring a LPS with truncated O antigen (19).

**Figure 6:**
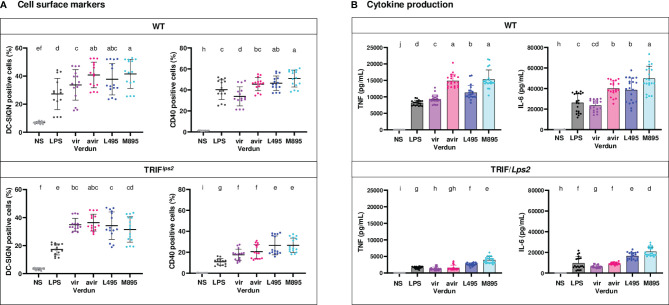
Contribution of the TRIF adaptor in the difference of BM-DCs activation upon stimulation with virulent compared to avirulent pathogenic strains of leptospires. Day 8 WT and TRIF/*Lps2* BM-DCs from C57BL/6 mice were stimulated with virulent and avirulent leptospires from the Verdun (Verdun vir and Verdun avir) and Manilae L495 (L495 and M895) strains at a Multiplicity of Infection (MOI) of 50 bacteria per cell. *E*. *coli* LPS (1 µg/mL) was used as a positive control for DCs stimulation. After 24h, cells were immunostained for the analysis of the expression of DC-SIGN and CD40 by flow cytometry (A, dot plot). TNF and IL-6 concentrations were also quantified in cell supernatants by ELISA assays (B, histograms with dot plot). Data were compared to results with non-stimulated cells (NS). Each dot represents technical triplicates **(A)** or quadruplicates **(B)** from two independent experiments with n=5 mice. Mean ± SD is also represented. Statistics indicated with lower-case letters allow for comparing, for each marker and each cytokine, all conditions within WT (upper panel) and TRIF/*Lps2* (lower panel). Conditions that share the same lower-case letters are not significantly different (p > 0.05). In contrast, conditions that do not share same letter(s) are significantly different.

At MOI 50, in WT BM-DCs, we observed significant differences in CD40 and DC-SIGN marker upregulations for the Verdun pair and only minimal differences for the Manilae pair, which were all lost in TRIF/*Lps2* BM-DCs (**Figure 6A**). Interestingly, both the virulent strains of Verdun Cl3 and L495 Manilae stimulated less cytokines production than their avirulent Verdun and M895 counterparts (**Figure 6B** and **Supplementary Figure 5B**). As seen in the TLR4^-/-^ BM-DCs, the cytokine levels were lower in TRIF/*Lps2* BM-DCs than in WT BM-DCs. Nevertheless, we observed, in TRIF/*Lps2* BM-DCs, a loss of the difference in TNF production between the Verdun strains. In contrast, the difference was conserved between Manilae strains. Moreover, the differences in IL-6 observed between both pairs of virulent and avirulent isolates were not totally abolished in TRIF/*Lps2* BM-DCs (**Figure 5B**). However, we observed in WT BM-DCs at MOI 50, for both pairs, a difference in IL-12p40 that was abolished in TRIF/*Lps2* BM-DCs (**Supplementary Figure 5B**).

**Figure 7:**
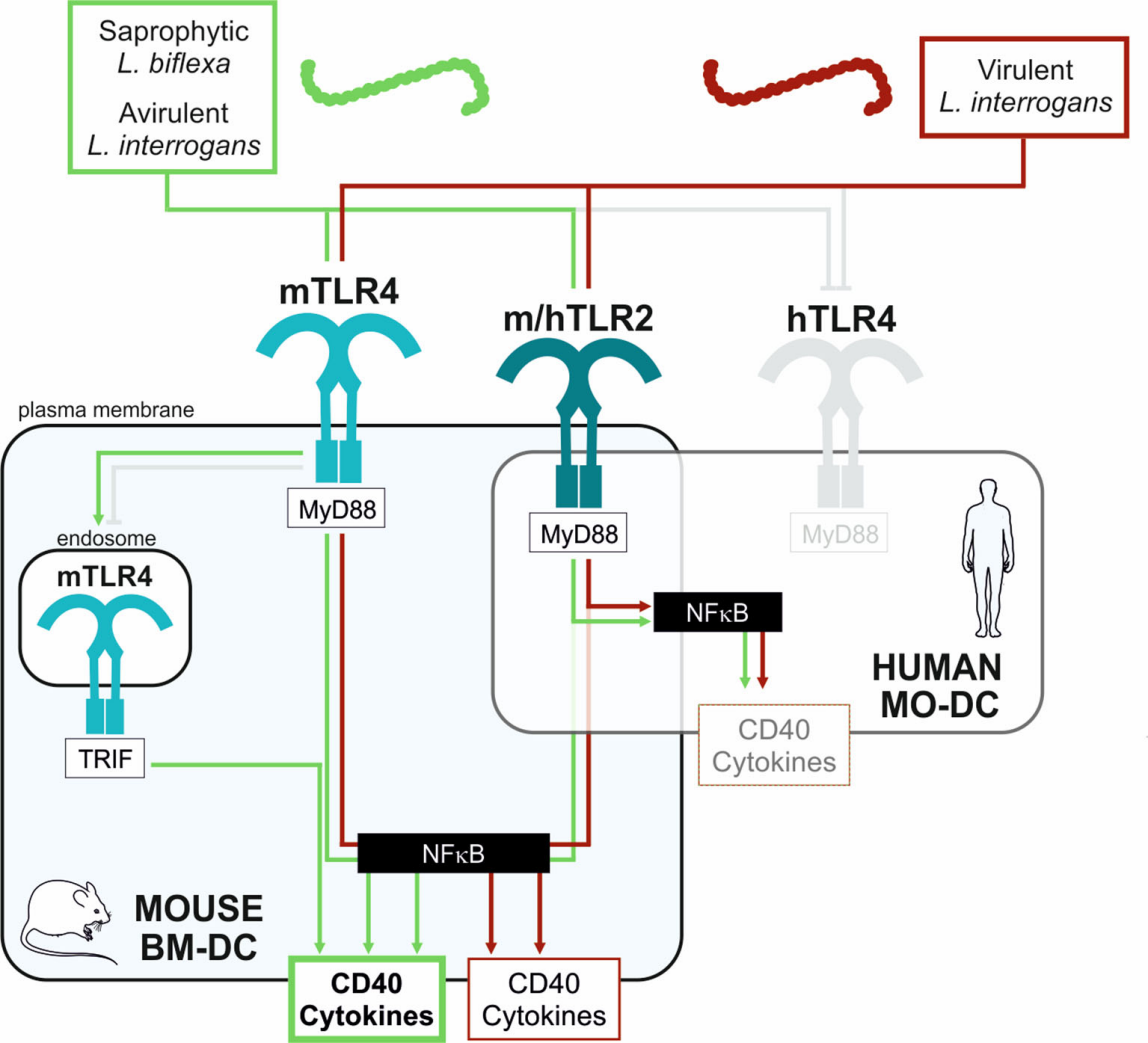
Schematic proposed model of mouse and human DCs activation upon stimulation with virulent compared to avirulent pathogenic strains of leptospires. Simplified model to explain the differential activation profiles in mouse (BM-DC, light blue frame) and human (MO-DCs, transparent frame) dendritic cells upon stimulation with leptospires. Saprophytic and avirulent strains of leptospires (green box) trigger a strong activation of mouse DCs since they activate 3 pathways: the murine (m)TLR4/MyD88, the endosomal mTLR4/TRIF, and the mTLR2/MyD88 pathways, all converging to the central NF-κB transcription factor, important for upregulation of activation markers (CD40) and cytokines production. In contrast, the virulent leptospires (red box) only stimulate 2 pathways in mouse DCs: the mTLR4/MyD88 and mTLR2/MyD88 pathways. The escape from the mTLR4/TRIF pathway by virulent leptospires (also evidenced in macrophages (20)) is depicted (grey capped line). Hence, the upregulation in CD40 and cytokines by virulent strains is lowered compared to avirulent strains. Finally, since leptospires are not recognized by the human (h)TLR4 (13, 14) (grey capped line), leptospires would activate the human DC mainly through the hTLR2/MyD88 pathway, which could explain both the low activation of DC by leptospires and the lack of differences between virulent and avirulent strains.

Altogether, in conclusion, these results suggest that pathogenic leptospires activate DC maturation, although less than saprophyte leptospires. Furthermore, compared to avirulent strains, we showed that virulent strains limit the activation of DCs by dampening the up-regulation of cell surface markers, such as DC-SIGN and CD40, as well as the production of inflammatory cytokines, all important to properly trigger the adaptive immune response and antibody production. This subversion of DC maturation is at least partially driven by TLR4 and TRIF signaling pathways. Consistent with these results, and by contrast to murine BM-DCs, we showed that human MO-DCs, which do not recognize leptospires through TLR4, were not able to discriminate between the virulent and avirulent strains of Verdun. These results are schematically represented in **Figure 7**.

## Discussion

### DC Models

In this work, we derived primary mouse bone marrow cells and human peripheral blood monocytes into immature DCs using GM-CSF, and GM-CSF and IL4, respectively. These established protocols aim at obtaining primary cell models for myeloid DCs, expressing high levels of CD11c and able to present antigens loaded onto MHC-II molecules. These cells express TLR receptors and produce cytokines upon infection with bacteria (23). Cytometry analysis showed that almost 97% of naïve cells derived from human monocytes were CD11c^+^ while only 42% (OF1) to 62% (B6) of bone marrow derived cells displayed the expected CD11c^+^/DC phenotype. This difference may be due to the step of monocyte purification among PBMCs. Therefore, cytokines quantified in cells supernatants in the human model correspond roughly to the actual production of MO-DCs. By contrast, in the mouse models, this quantification reflects cytokine production by both BM-DCs and other cells, such as macrophages. However, in BM-DCs, the dose-dependent stimulation with different leptospires equally modulated cytokines and activation markers, only present at the surface of CD11c^+^ cells, suggesting that the measured cytokines were produced mostly by BM-DCs. Finally, we observed in BM-DCs a downregulation of CD11c^+^ upon LPS stimulation. These data were consistent with the literature (22), and the high cytokine production and cell surface activation are strong evidence for proper DCs maturation.

### Leptospires Activate Primary Human and Mouse DCs

To the best of our knowledge, only one study has investigated the interaction of leptospires with DCs. In that work, incubation for 48 h with paraformaldehyde fixed *L. biflexa* or different serovars of *L. interrogans* all activated human MO-DCs, with no differences between serovars in upregulation of CD83 and CD86 co-stimulation markers. However, differences in IL-12p70, IL-6 and IL-10 secretion were described among serovars, with a tendency of a less virulent strain of Autumnalis isolated from a patient who recovered to better activate cytokine secretion compared to the one isolated from a deceased patient (16).

The present study is consistent with these findings and showed that *L. biflexa* and different serovars of *L. interrogans*, either live or heat-inactivated, equally activated human MO-DCs in terms of expression of HLA-DR, and co-stimulation markers CD83 and CD86. However, cytokine production showed differences according to serovars, with Patoc and Manilae triggering more cytokines than Icterohaemorrhagiae. Interestingly, this pattern of cytokines secretion was also found in mouse DCs, using two different backgrounds of mice, the inbred C57BL6/J mice and originally outbred OF1 mice, and without differences when using male- or female-derived cells. In contrast to human MO-DCs, we found that the extent of marker expression at the surface of mouse BM-DCs was also serovar-dependent. We observed a dose-dependent increase in expression of co-stimulation molecules CD40 and CD80, as well as MHC-II markers, with the same differences as noted for the cytokines, with Patoc and Manilae activating better than Icterohaemorrhagiae. Moreover, if live and heat-killed leptospires upregulated the CD40 markers and production of TNF, IL-6, IL-12p40 with no marked differences, MHC-II and CD80 upregulation were decreased or lost with heat-killed *L. interrogans.* These results suggest that the serovar-specific pattern of DC activation does not depend on the host, but rather on leptospiral MAMPs. On the one hand, we may hypothesize that, for example, sugar components of O antigens of LPS that are known to differ greatly between serovars (24, 25), and are heat-resistant, could be involved in the CD40 upregulation and production of cytokines cited above. Of note, upregulation of CD40 by *E. coli* LPS was the highest observed among the different co-stimulation markers we studied. On the other hand, a heat-sensitive component could be involved in MHC-II and CD80 expression, as was shown for rotavirus or Poly-IC that both upregulated those markers in mouse B6 BM-DCs, through TLR3 signaling (26). In our case, it could be due to bacterial messenger RNA, recognized by TLR7/8, and considered as a marker of bacterial viability (27). Paradoxically, it has been shown that *Brucella abortus* RNA along with its lipoproteins contributed to downregulation of the expression of MHC-II induced by IFN-γ on mouse monocytes/macrophages (28). This may prompt us to further study the potential modulatory role of *Leptospira* mRNA in evasion from immune responses.

### DC-SIGN

The C-type lectin DC-SIGN is a specific marker of DCs recognizing mannose-containing carbohydrates. DC-SIGN is also involved in adhesion of DC to T cells and is required for DC-induced proliferation of resting T cells (29). We investigated the presence of DC-SIGN at the surface of *Leptospira*-exposed DCs. We found dose-dependent upregulation of DC-SIGN in BM-DCs after *Leptospira* infection. The activation was equivalent between serovars, in contrast to the activation of co-stimulation molecules or MHC-II. However, in OF1 BM-DCs, levels of DC-SIGN upregulation were lower upon stimulation with LPS, L495 and Patoc than in B6 BM-DCs, and Verdun vir did not upregulate DC-SIGN. We could not draw any conclusions about MO-DCs, since we did not find upregulation of this marker with leptospires or LPS, despite using the same antibody targeted at human DC-SIGN that worked for both BM-DCs. Nevertheless, using inactivated leptospires, Gaudart et al. (16) demonstrated *in vitro* the binding of saprophytic *L. biflexa* as well as 22 serovars of *L. interrogans* to the human DC-SIGN, and showed DC-SIGN expression on uninfected MO-DCs. Our study further showed that leptospires upregulated DC-SIGN expression in mouse DCs. Moreover, it has been shown that PBMCs from volunteers stimulated *in vitro* with *Leptospira* activated T cell proliferation (30). These data suggest that leptospires are able to induce T-cell activation. However, whether *Leptospira*-activated DCs could induce T cell activation and proliferation remains an important unanswered question.

Interestingly, other spirochetes such as *Borrelia* B31 are phagocytosed by, and also weakly activate, MO-DCs with up-regulation of CD80, CD83, CD40, HLA-DR markers and production of IL-8 (31). It was further shown that tick saliva inhibits DC activation through the Salp1 protein binding to DC-SIGN. In addition, *Treponema pallidum* patients with secondary syphilis express more DC-SIGN on dermal and circulating DCs than controls (32). Moreover, DC-SIGN binds lipoarabinomannans of *Mycobacteria*, which inhibits DC activation and enhances IL-10 (33). Whereas the binding to DC-SIGN could be a strategy used by leptospires to dampen the DC activation and enhance the expression of IL-10, an anti-inflammatory cytokine that has been shown to be instrumental for successful infection by leptospires and renal colonization (34, 35), remains to be characterized.

### Nonvirulent Leptospires Activate Better Than Parental Virulent *L. Interrogans*

An interesting feature highlighted in our study is the observation that nonvirulent strains of pathogenic leptospires tend to better activate mouse BM-DCs than the parental virulent strains, which was not observed in human MO-DCs. This observation is striking and proved true for different serovars and strains. Indeed, for logistical reasons, we compared in this study two different sets of Icterohaemorrhagiae Verdun virulent and attenuated strains, independently obtained after extensive *in vitro* passages (strains Verdun Cl3/Verdun Cl3 p104 (10) and Verdun Vir/avir used at IPNC for diagnostic of leptospirosis by Micro Agglutination Test. We also compared the virulent Manilae L495 to M895, a defined avirulent LPS mutant strains obtained by random mutagenesis (19). A better activation of DCs is supposed to drive a better activation of T cells and a better B cell response with production of neutralizing antibodies. This potentially could explain the lack of virulence of the saprophytic strain or attenuated strains that do not provoke disease nor colonize their hosts (10, 19, 36). Interestingly, we have recently studied the long-term humoral antibody response after infection of B6 mice with both sets of Manilae L495/M895 and Icterohaemorrhagiae Verdun Cl3/Verdun Cl3p104 (10). Despite the finding of an association of a sustained IgM production with chronic renal colonization that was not observed with the Verdun Cl3, we found a limited production of specific Immunoglobulins G (IgG) after infection with Verdun Cl3 compared to Manilae L495 virulent or the M895 avirulent mutant that did not colonize either (10). Therefore, the finding that Icterohaemorrhagiae resulted in lower activation of DCs than Manilae serovar could fit the hypothesis that the former would limit the T cell activation and B cell antibody production. However, for both serovars, all subtypes of IgGs were equivalent or decreased with the mutant compared to the parental strains (10), making this hypothesis difficult to sustain. Therefore, the functional consequences of the limitation of DC activation by pathogenic *L. interrogans* remain to be elucidated.

### TLR4

Interestingly, it was shown that TLR4 was crucial to inducing maturation of mouse splenic DCs with classical *E. coli* LPS (37). A recent study showed that in C3H/HeJ mice deficient for TLR4, infection with the pathogenic *L. interrogans* Copenhageni strain Fiocruz L1-130 increased the number of DCs in spleens (38), showing that *in vivo* in mice, leptospires can trigger DC activation independently of TLR4. This situation is reminiscent of what we found in MO-DCs. Although they are not immune-compromised, human MO-DCs do not sense leptospires through TLR4, and yet we measured up-regulation of the markers and cytokines upon infection with *Leptospira*. We also found in BM-DCs that except for CD40, the other activation markers, as well as DC-SIGN, did not rely on TLR4 signaling, whereas cytokine secretion was decreased in TLR4^-/-^ BM-DCs. Interestingly, we found that the TLR4-dependent differences between virulent and avirulent mutants in CD40 and TNF secretion were also TRIF dependent, whereas the difference in IL-6 was not, and therefore might depend on Myd88, the other arm of the TLR4 response. We can speculate that MO-DCs would produce fewer cytokines than if leptospires could stimulate through TLR4. Of note, the decrease in TLR4^-/-^ BM-DCs of CD40 was not complete with the LPS we used. This LPS “EB” from InvivoGen is only partly purified and has some TLR2 activity. Since we showed in bone marrow derived macrophages that part of the leptospiral activation was TLR2 dependent (13), it is possible that the observed activation of MO-DCs by leptospires could also be dependent on TLR2.

Unexpectedly, we found that the difference in DC-SIGN activation between virulent and avirulent strains also depended on TRIF, which is usually associated with TLR4 and TLR3, or less frequently, with TLR2 signaling (39). Since heating leptospires did not change the DC-SIGN activation profile, it could suggest that the dampening of DC-SIGN activation by virulent strains may be due to leptospiral lipoproteins binding to TLR2 (40). Indeed, lipoproteins are highly abundant in pathogenic leptospires and are untypically heat-resistant. Moreover, leptospiral lipoproteins are known to be involved in immune evasion (41, 42).

Interaction between CD40 on B cells and DCs and CD40L on T cells is responsible for several aspects of acquired immune responses, including the generation of memory B cells crucial for long-term resistance to pathogens. It was shown that no memory T cells were found among PBMCs from patients who recovered from leptospirosis (30). We showed a dampening of CD40 upregulation by virulent strains in BM-DCs, but since we failed to measure the convincing expression of CD40 in human MO-DCs, the question of whether the lack of human T and B cell memory response is linked to the lack of TLR4 sensing of leptospiral LPS and activation of CD40 in humans remains to be elucidated.

### Role of LPS

Of note, other bacteria, such as *Brucella melitensis*, also possess an atypical LPS, which shows diminished activation of human MO-DCs and murine BM-DCs, and poorly activates T-cell proliferation (37). Strikingly, however, a LPS mutant of *B. melitensis*, devoid of a lateral oligosaccharide linked to the core section, enhances the BM-DCs’ secretion of IL-6, TNF and IL-12p40 cytokines, and upregulation of CD40 and CD86 in a TLR4 dependent manner. That study suggested that a defective activation of DC *in vivo* may contribute to the immune system avoidance elicited by *Brucella*, favoring the establishment of chronic disease (37). Likewise, our work suggests a similar strategy of pathogenic leptospires to avoid DC activation through peculiar characteristics of their LPS O antigen that might not be sensed by the TLR4/TRIF signaling, as we recently showed in macrophages (20). Interestingly, the most striking results were obtained with highly passaged Verdun strains, which compared to the parental Verdun strains also present differences in silver stained LPS profiles, with a shorter O antigen part (**Supplementary Figure 6**), likewise observed with M895 (19, 20). These Verdun avirulent strains presenting reduced size of O antigen may also harbor other uncharacterized mutations that remain to be studied, also participating in the limitation of DC activation. The structure of leptospiral lipid A has been deciphered and data show that it is conserved between different serovars (43, 44), it would thus be of interest to solve the precise structure of the carbohydrate component of the core and O antigen of leptospiral LPS. The LPS mutant M895 strain that we recently showed to induce the TLR4/TRIF pathways (20), has been together with the M1352, another LPS mutant, proposed as live vaccines (18, 45). The present findings showing the increased ability of the M895 LPS mutant to activate dendritic cells most probably contributes to its attenuated phenotype and protective effect.

Altogether, we showed *in vitro* that compared to saprophytic species, pathogenic leptospires limit DC activation in both human and mouse DCs. Our data suggest an important role of the leptospiral LPS of pathogenic leptospires in the dampening of CD40 expression and cytokine secretion through TLR4 and TRIF pathways. In addition, we also showed limitations of DC-SIGN and MHC-II expression through unknown leptospiral components. In conclusion, this study highlighted a novel immune evasion mechanism of pathogenic *Leptospira*.

## Data Availability Statement

The original contributions presented in the study are included in the article/**Supplementary Material**. Further inquiries can be directed to the corresponding author.

## Ethics Statement

The studies involving human participants were reviewed and approved by New Caledonian local ethic committee. Authorization was obtained for the use of human leucocytes from healthy platelet- donors after cytapheresis at the Territorial Hospital Center in New Caledonia. All methods and all experimental protocols were carried out in accordance with relevant human ethical guidelines and regulations of the Institut Pasteur of New Caledonia (IPNC). The donors (of both sexes) consented that their blood will be used for research. The patients/participants provided their written informed consent to participate in this study. The animal study was reviewed and approved according to the guidelines of the Animal Care and Use Committees of the Institut Pasteur and followed European Recommendation Directive 2010/63/EU. Experiments with mice were carried out under the protocol number HA0036, approved by the Institut Pasteur ethics committee (CETEA 89) (Paris, France). All experiments have been performed on animal cells of both sexes.

## Author Contributions

Conceptualization and Initial experiments: MM, CW. Methodology and experiments on MO-DCs and BM-DCs/OF1: JC. C57BL/6J experiments: FV-P. Figures and legends: JC, FV-P. Validation and data analysis: JC, FV-P, CW, MM. Statistical analysis: JC. Manilae LPS mutants; BA, GM. Supervision and project administration: CW. Funding acquisition and resources: CW, MM. Initial draft preparation: CW. Writing Intro/Mat/Met: JC, FV-P. Review and Editing: all authors. English editing: BA. All authors have read and agreed to the published version of the manuscript.

## Funding

This research was funded by the “Programme Transversal de Recherche” (PTR 66-2017) from the Institut Pasteur de Paris, the French Ministry of Higher Education, Research and Innovation (MESRI), the Institut Pasteur de Nouvelle Calédonie (IPNC) and the Pasteur Network. We are thankful to the Programme Calmette and Yersin and the Pasteur Network for the scholarships accorded to MM and JC for travelling mission to IPP. MM position is financed by the Government of New Caledonia. Postdoc position of JC was supported by the Government of New Caledonia and the IPNC. Work at Monash University was supported by the Australian Research Council and the National Health and Medical Research Council.

## Conflict of Interest

The authors declare that the research was conducted in the absence of any commercial or financial relationships that could be construed as a potential conflict of interest.

## Publisher’s Note

All claims expressed in this article are solely those of the authors and do not necessarily represent those of their affiliated organizations, or those of the publisher, the editors and the reviewers. Any product that may be evaluated in this article, or claim that may be made by its manufacturer, is not guaranteed or endorsed by the publisher.

## Abbreviations

FBS, Fetal Bovine Serum; IPP, Institut Pasteur de Paris, IPNC, Institut Pasteur de Nouvelle Calédonie; IMDM, Iscove’s Modified Dulbecco’s Media; RPMI, Roswell Park Memorial Institute; NEAA, Non-Essential Amino Acids; rhGM-CSF, recombinant human granulocyte-macrophage colony stimulating factor; rhIL-4, human recombinant interleukin-4; rmGM-CSF, recombinant mouse Granulocyte-Macrophage Colony-Stimulating Factor.
