# Supplementary material for: Pathogenic Leptospires Limit Dendritic Cell Activation Through Avoidance of TLR4 and TRIF Signaling
Source: Front Immunol. 2022 Jun 22;13:911778. doi: 10.3389/fimmu.2022.911778 (PMC9258186; doi:10.3389/fimmu.2022.911778)
Supplement: Supplementary file 1 [file DataSheet_1.pdf]

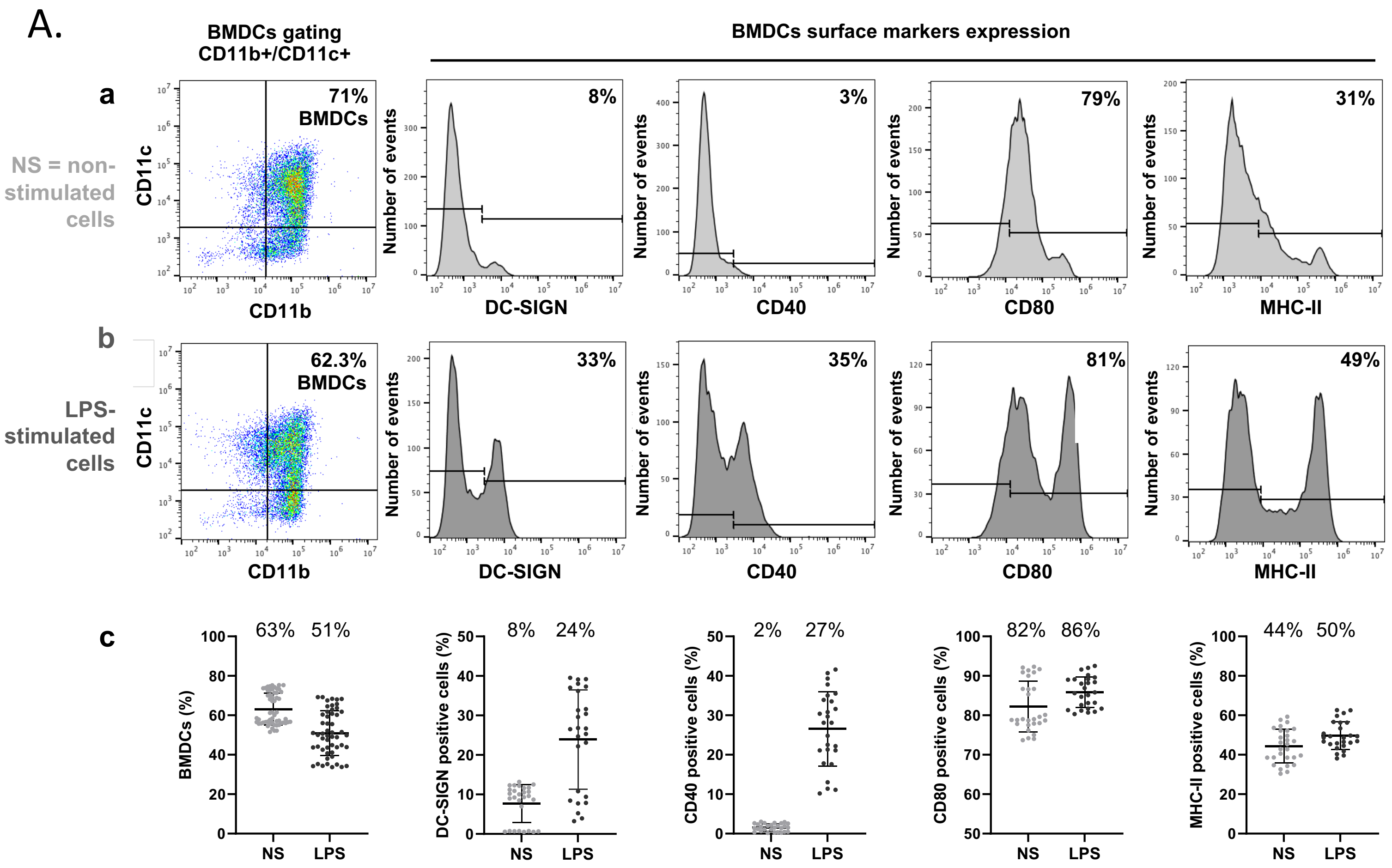

Supplementary Figure 1 : Characterization of the derived Dendritic Cells models: Gating strategy, LPS activation and cell surface markers. Mouse bone marrow progenitor cells were cultured for 8 days in the presence of rmGM-CSF and were stimulated with *E. coli* LPS, 1 µg/mL for 24h. After stimulation, cells were immunostained for lineage (CD11c and CD11b) and activation (DC-SIGN, CD40, CD80 and MHC-II) cell surface markers. Percentage of cells positive for a given marker was analyzed by flow cytometry as a reflection of the level of expression of each marker and data were compared to results with non-stimulated cells (NS).

Supplementary Figure 1A – Bone marrow derived Dendritic Cells (BM-DCs) from C57BL/6 mice. a and b : Dot plots show the gating strategy for the selection of CD11b+/CD11c+ BM-DCs from C57BL/6 mice, either non stimulated (NS, a) or stimulated with LPS (b). Percentage of selected BM-DCs is indicated (see upper right corner). Fluorescence histograms show the intensity of DC-SIGN, CD40, CD80 and MHC-II staining and the corresponding number of events in the gated CD11b+/CD11c+ BM-DCs population. Percentage of positive cells is indicated for each marker. The black lines show threshold for CD11b+, CD11c+, DC-SIGN+, CD40+, CD80+ and MHC-II+ cells, determined using control isotype staining. Figures in panels a and b are representative of one stimulation replicate among five independent experiments with n=6 mice. **c** : Dot plot diagrams show the mean percentage of CD11b+/CD11c+ BM-DCs and the mean percentage of DC-SIGN+, CD40+, CD80+ and MHC-II+ among the gated BM-DCs obtained from five independent experiments with each dot representing a stimulation triplicate. Mean ± standard deviation (SD) is also represented (black lines).

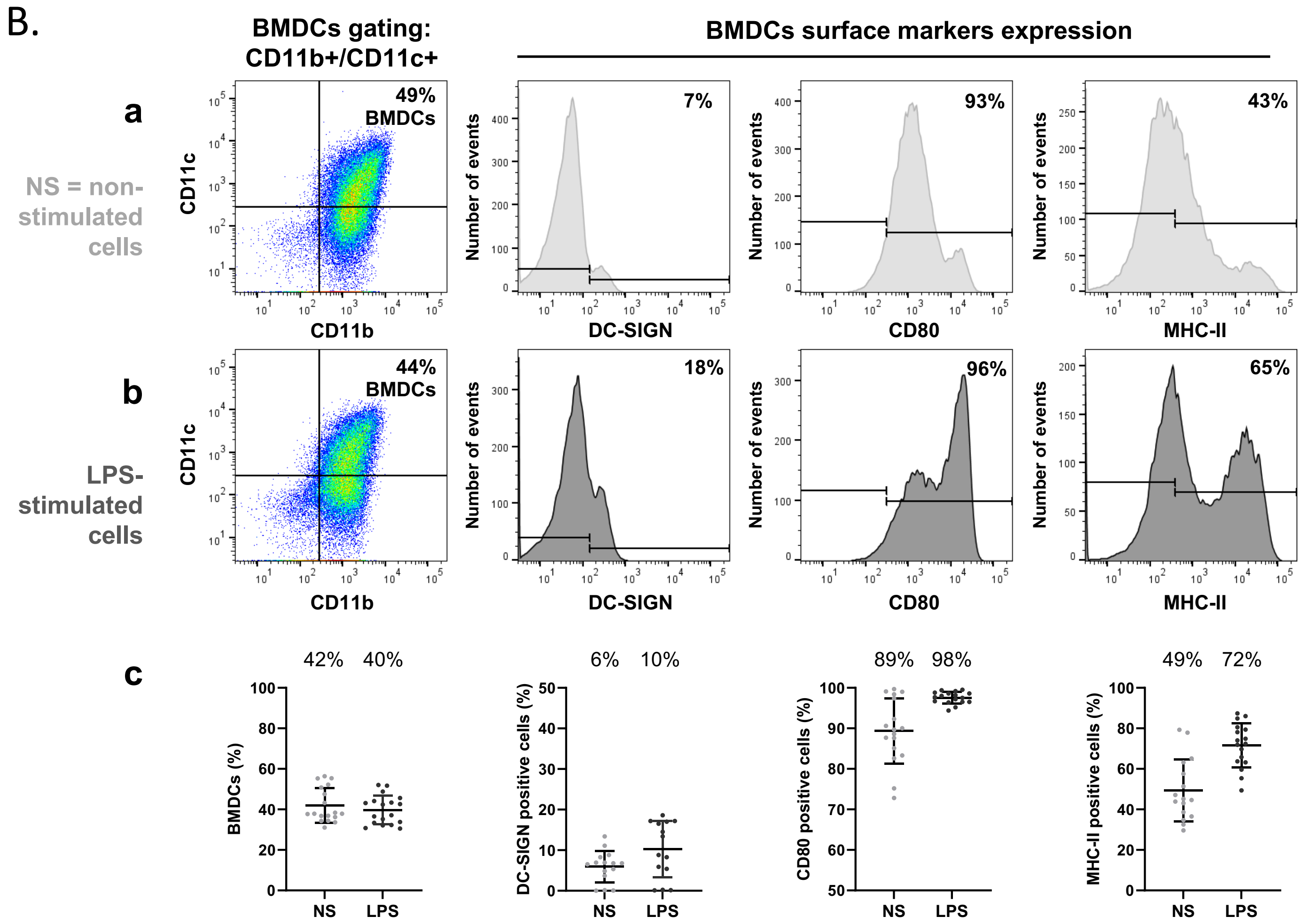

Supplementary Figure 1B - Bone marrow derived Dendritic Cells (BM-DCs) from OF1 mice. **a and b** : Dot plots show the gating strategy for the selection of CD11b+/CD11c+ BM-DCs from OF1 mice, either non stimulated (NS, a) or stimulated with *E. Coli* LPS (b). Percentage of selected BM-DCs is indicated (see upper right corner). Fluorescence histograms show the intensity of DC-SIGN, CD80 and MHC-II staining and the corresponding number of events in the gated CD11b+/CD11c+ BM-DCs population. Percentage of positive cells is indicated for each marker. The black lines show threshold for CD11b+, CD11c+, DC-SIGN+, CD80+ and MHC-II+ cells, determined using control isotype staining. Figures in panels a and b are representative of one stimulation triplicate among six independent experiments with n=6 mice. **c** : Dot plot diagrams show the mean percentage of CD11b+/CD11c+ BM-DCs and the mean percentage of DC-SIGN+, CD40+, CD80+ and MHC-II+ among the gated BM-DCs obtained from six independent experiments with each dot representing a stimulation triplicate from n=3 to 6 mice. Mean ± standard deviation (SD) is also represented (black lines).

C.

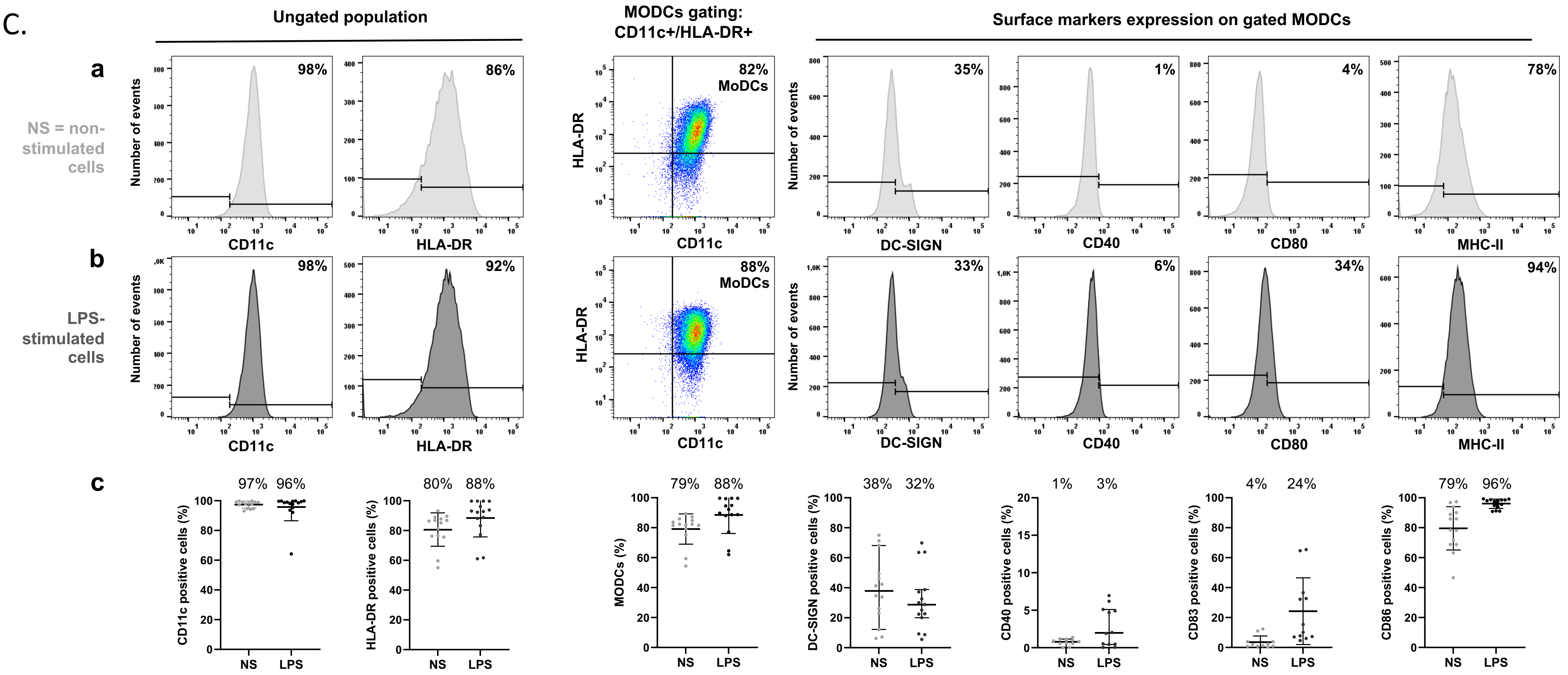

Supplementary Figure 1C - Human Monocyte derived Dendritic Cells (MO-DCs) model. Monocytes purified from PBMCs were cultured for 8 days in the presence of rhGM-CSF and rhIL-4 and were stimulated with LPS (1 µg/mL) for 24h. After stimulation, cells were immunostained for CD11c and HLA-DR (lineage markers) and DC-SIGN, CD40, CD83 and CD86 (activation markers). Percentage of cells positive for a given marker was analyzed by flow cytometry as a reflection of the level of expression of each marker and data were compared to results with non-stimulated cells (NS). **a and b**: Histograms show the intensity of CD11c and HLA-DR staining and the corresponding number of events in the ungated population. Dot plots show the gating strategy for the selection of CD11c+/HLA-DR+ Monocyte derived DCs (MO-DCs) either non-stimulated (a) or stimulated with LPS (b). Percentage of selected MO-DCs is indicated (see upper right corner). Histograms show the intensity of DC-SIGN, CD40, CD83 and CD86 staining and the corresponding number of events in the gated CD11c+/HLA-DR+ MO-DCs population. The HLA-DR staining on the ungated population is also represented. Percentage of positive cells is indicated for each marker. The black lines show threshold for CD11c+, HLA-DR+, DC-SIGN+, CD40+, CD83+ and CD86+ cells, determined using control isotype staining. Figures in panels a and b are representative of one stimulation triplicate among five independent blood donors. **c** : Dot plot diagrams show the mean percentage of CD11b+/CD11c+ BM-DCs and the mean percentage of DC-SIGN+, CD40+, CD80+ and MHC-II+ among the gated BM-DCs obtained from five independent donors with each dot representing a stimulation triplicate. Mean ± standard deviation (SD) is also represented (black lines).

D.

a – OF1 BM-DCs **b – MO-DCs**

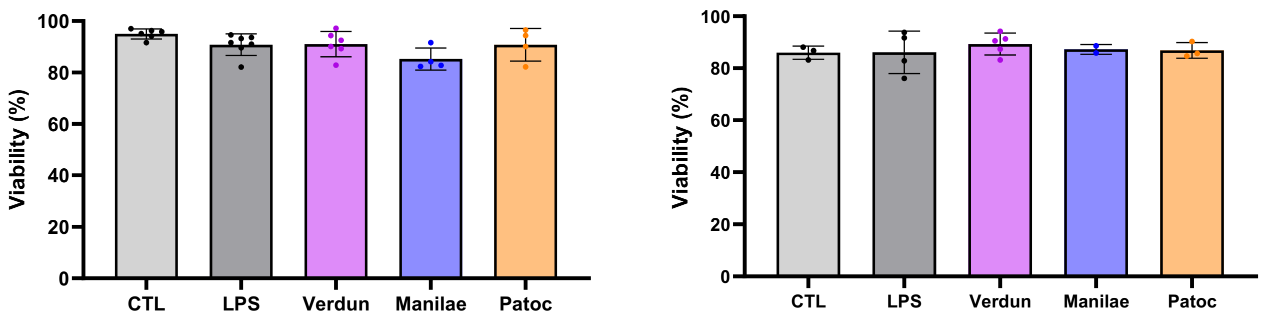

Supplementary Figure 1D: exposure to *Leptospira* does not alter DCs' viability. Day 8 BM-DCs from OF1 mice (**a**) and MO-DCs derived from human monocytes (**b**) were stimulated for 24h with Verdun vir, Manilae L495 and Patoc leptospires at a Multiplicity of Infection (MOI) of 100 bacteria per cell, or with *E. coli* LPS (1 µg/mL). After stimulation, total cells were stained with the eFluor 780 permeability dye. Positivity threshold was determined with unstained cells. $eFluor^{+}$ cells were gated as the dead cells and percentage of viable cells (viability) is shown in histograms with dot plot. Each dot represents stimulation replicates from two to four independent experiments.

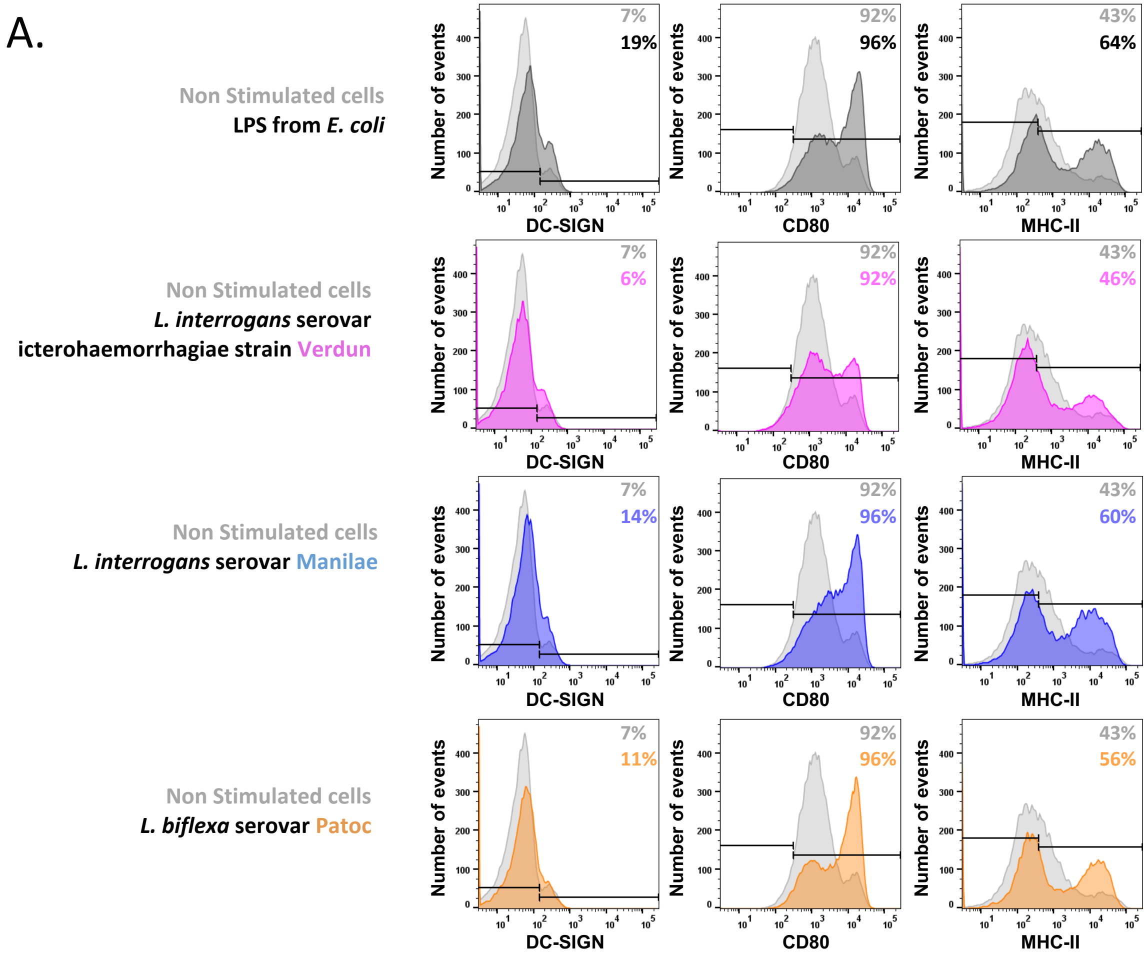

Supplementary Figure 2 : Exposure to leptospires induces the activation of BM-DCs from OF1 mice
Supplementary Figure 1A : Day 8 BM-DCs derived from OF1 mice were stimulated with Verdun vir, Manilae L495 and Patoc leptospires at a Multiplicity of Infection (MOI) of 100 bacteria per cell, or with *E. coli* LPS (1 µg/mL) for 24h. After stimulation, cells were immunostained for the analysis of the expression of DC-SIGN, CD80 and MHC-II through flow cytometry. Histograms show the intensity of DC-SIGN, CD80 and MHC-II staining versus the number of events in the gated CD11b+/CD11c+ BM-DCs population. Data are comparted to histograms from non-stimulated cells. Percentage of positive cells is indicated for each marker. The black lines show threshold for CD11b+, CD11c+, DC-SIGN+, CD80+ and MHC-II+ cells, determined using control isotype staining. Figures are representative of one stimulation replicate among five independent experiments with n=4 to 5 mice.

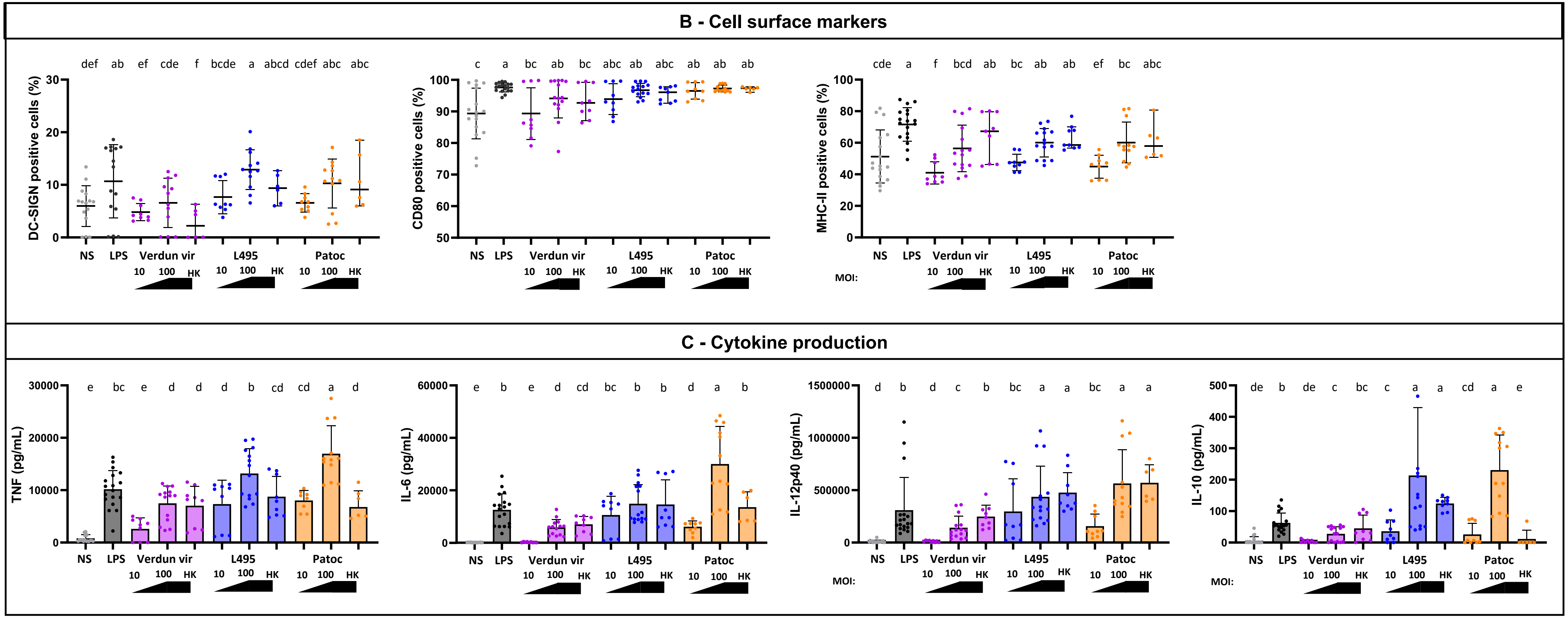

Supplementary Figure 2: Exposure to leptospires induces the activation of BM-DCs from OF1 mice

Supplementary Figure 2B

Day 8 BM-DCs derived from OF1 mice were stimulated with either live of heat-killed (HK) Verdun vir, Manilae L495 and Patoc leptospires at a Multiplicity of Infection (MOI) of 100 bacteria per cell, or with *E. coli* LPS (1 µg/mL) for 24h. Data are represented in dot plot diagrams showing the percentage of BM-DCs positive for DC-SIGN, CD80 and MHC-II staining. Each dot represents stimulation triplicates from four independent experiments with n=3 to 4 mice. Mean ± SD is also represented (black lines). Conditions with the same lower-case letters are not significantly different ($p > 0.05$) using Kruskal-Wallis comparison test followed by post-hoc Fisher's LSD test. NS : non-stimulated cells.

Supplementary Figure 2C

Cytokine concentrations were quantified in cell supernatants from BM-DCs in Sup Fig 3B. Results are represented in histograms with dot plot showing the concentration of pro-inflammatory TNF, IL-6 and IL-12 and anti-inflammatory IL-10. Each dot represents stimulation triplicates from four independent experiments with n=3 to 4 mice. Mean ± SD is also represented. Conditions with the same lower-case letters are not significantly different ($p > 0.05$) using Kruskal-Wallis comparison test followed by post-hoc Fisher's LSD test. NS : non-stimulated cells.

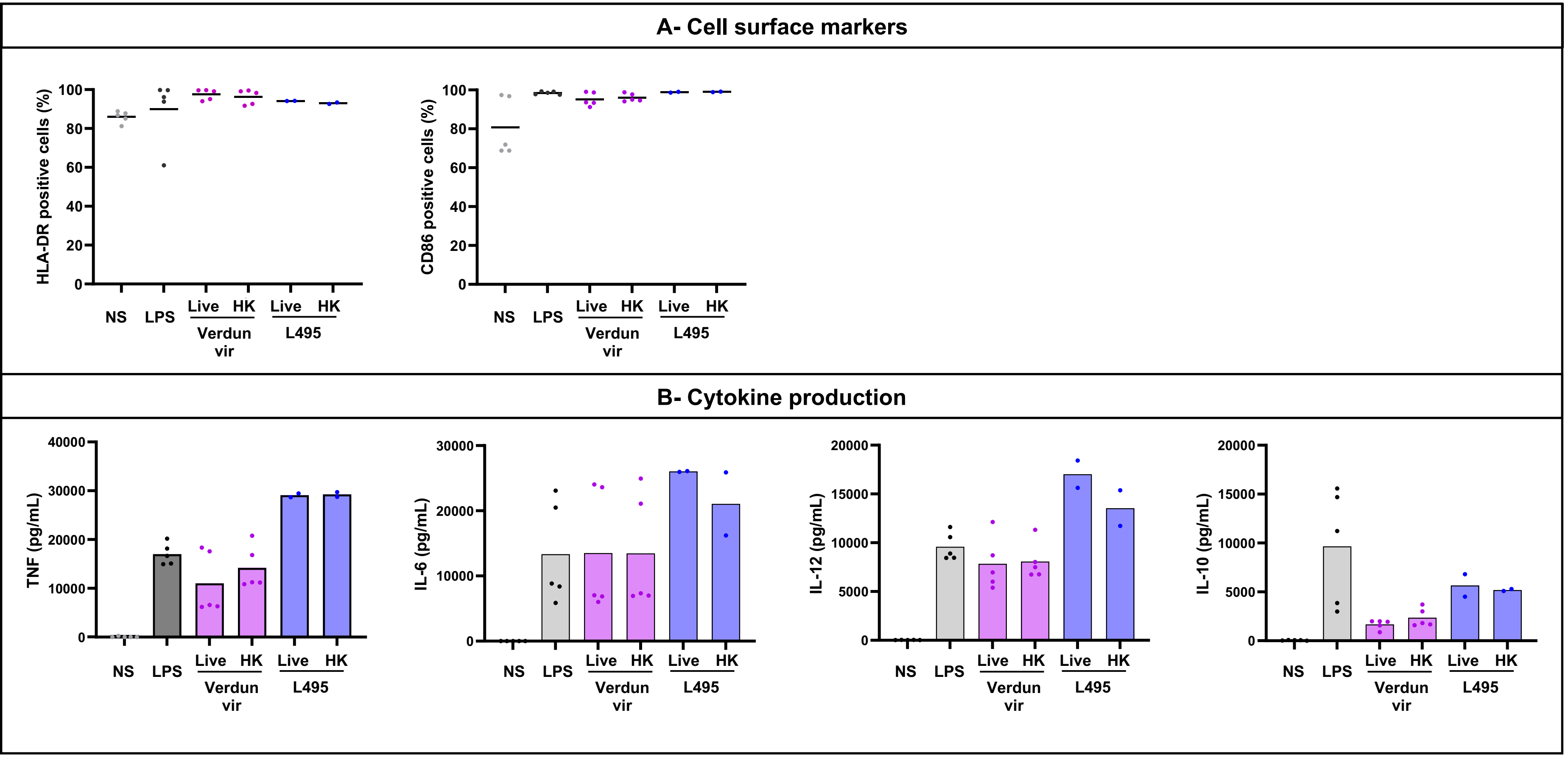

Supplementary Figure 3 : Exposure to leptospires, whether live or heat-killed, activates human MO-DCs

Day 8 Human MO-DCs were stimulated with either live or Heat-Killed (HK) Verdun vir, Verdun avir, Manilae L495 and Patoc leptospires at a Multiplicity of Infection (MOI) of 100 bacteria per cell for 24h. *E. coli* LPS (1 µg/mL) was used as a positive control for DCs stimulation. After 24h, cells were immunostained for the analysis of the expression of **A-** cell surface markers through flow cytometry and **B-** cytokine concentrations were also quantified in cell supernatants by ELISA assays. Data were compared to results with non-stimulated cells (NS).

A : Dot plot diagrams show the percentage of ungated cells positive for HLA-DR staining as well as the percentage of MO-DCs positive for CD86. **B:** Histograms with dot plot show the concentration of pro-inflammatory TNF-a, IL-6 and IL-12 and anti-inflammatory IL-10 in cell supernatants.

Each dot represents a stimulation replicates from one (L495) to two (Verdun vir, NS and LPS) blood donors. Mean is also represented. For each panel, conditions with the same lower-case letters are not significantly different ($p > 0.05$) using Kruskal-Wallis comparison test followed by post-hoc Fisher's LSD test.

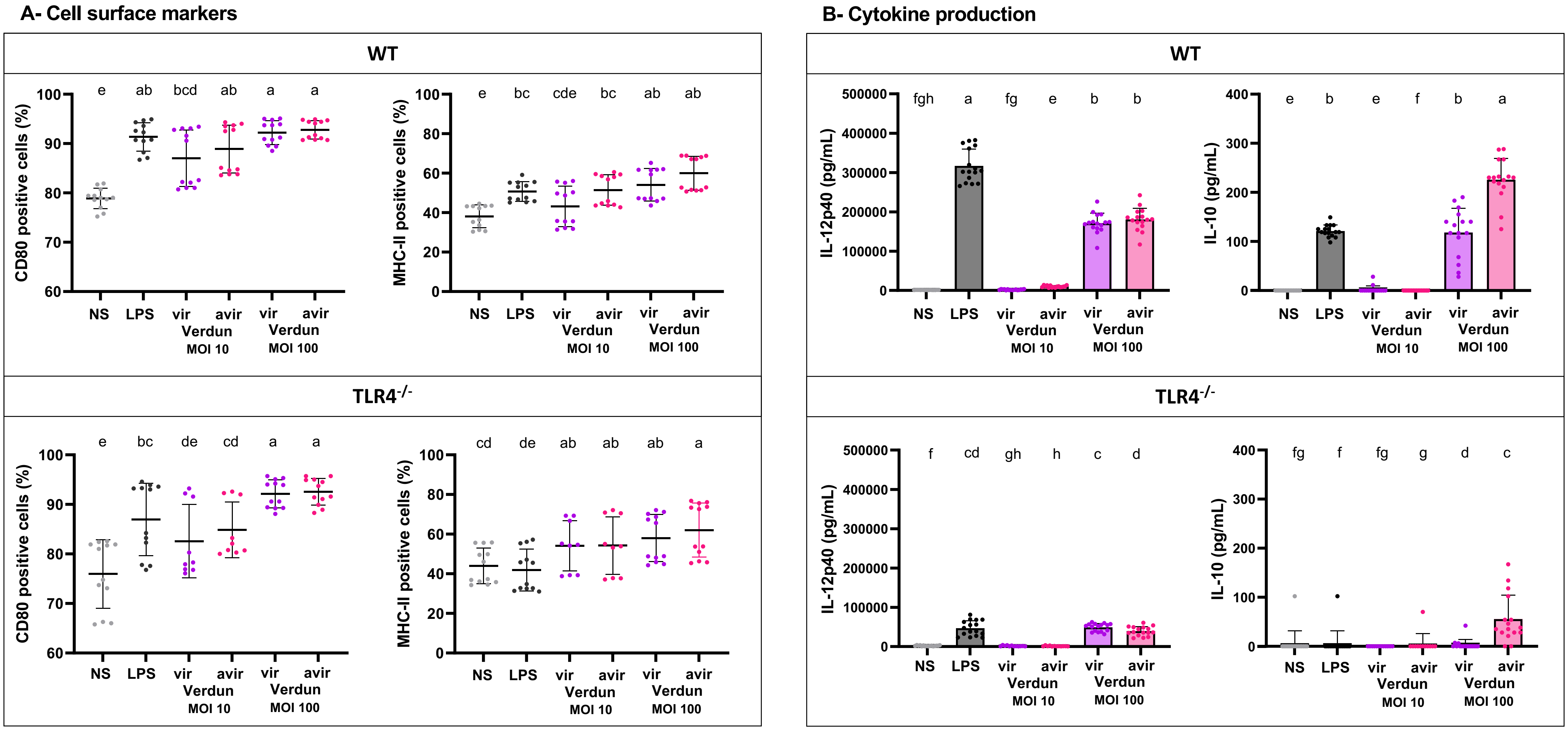

Supplementary Figure 4: Contribution of TLR4 in the difference of BM-DCs activation upon stimulation with virulent compared to avirulent pathogenic strains of leptospires.
Day 8 WT and TLR4$^{-/-}$ BM-DCs from C57BL/6 mice were stimulated with virulent and avirulent leptospires from the Verdun (Verdun vir and Verdun avir) strains at a Multiplicity of Infection (MOI) of 10 or 100 bacteria per cell. *E. coli* LPS (1 µg/mL) was used as a positive control for DCs stimulation. **A**: After 24h, cells were immuno-stained for the analysis of the expression of CD80 and MHC-II through flow cytometry and percentage of positive cells are represented in dot plot. **B**: IL-12p40 and IL-10 concentrations were also quantified in cell supernatants by ELISA assays and results are displayed in histograms with dot plot. Data were compared to results with non-stimulated cells (NS). Each dot represents technical triplicates (A) or quadruplicates (B) from 2 independent experiments with n=4 mice. Mean ± SD is also represented. Statistics indicated with lower-case letters allow to compare, for each marker and each cytokine, all conditions within WT (upper panel) and TLR4$^{-/-}$ (lower panel). Conditions with the same lower-case letters are not significantly different ($p > 0.05$) using Kruskal-Wallis comparison test followed by post-hoc Fisher's LSD test.

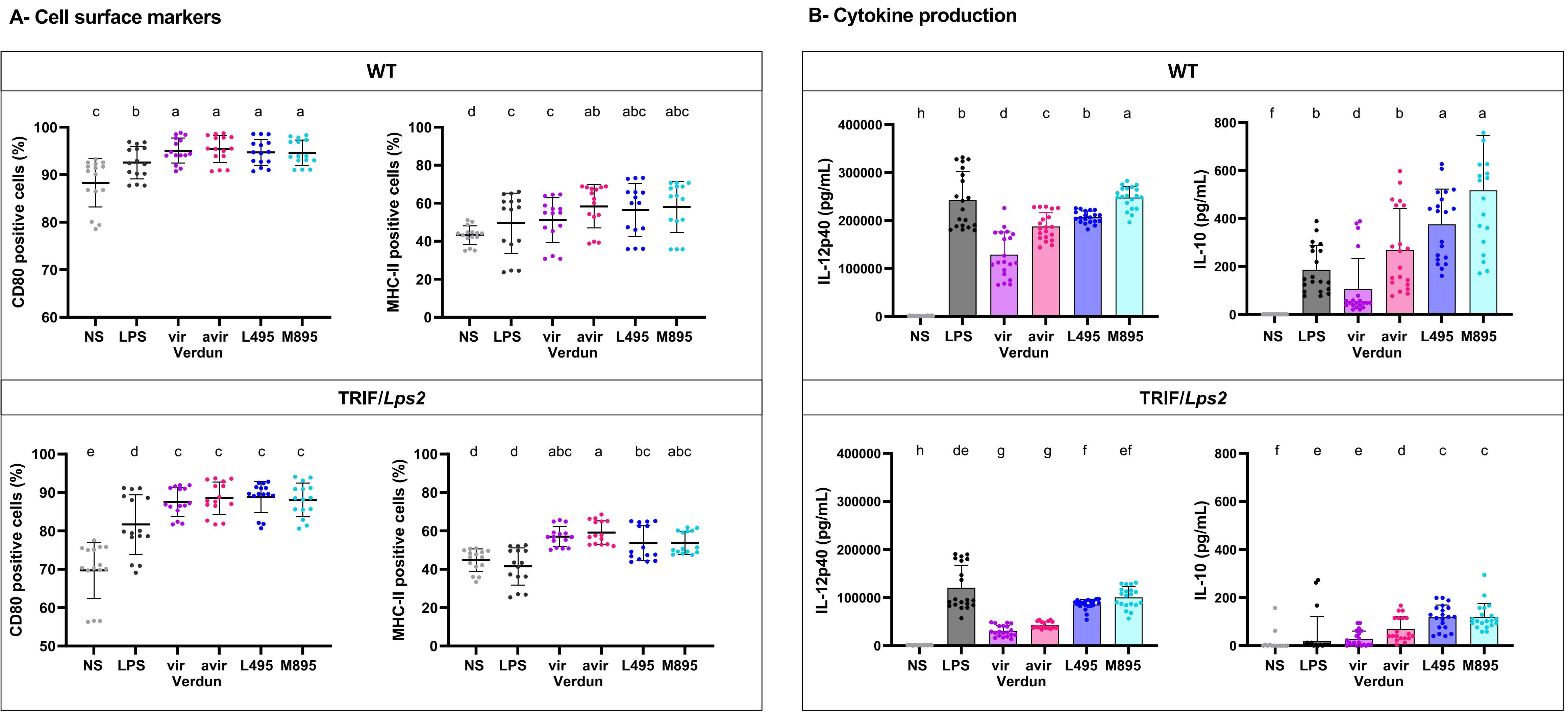

Supplementary Figure 5 : Contribution of the TRIF adaptor in the difference of BM-DCs activation upon stimulation with virulent compared to avirulent pathogenic strains of leptospires

Day 8 WT and TRIF/*Lps2* BM-DCs from C57BL/6 mice were stimulated with virulent and avirulent leptospires from the Verdun (Verdun vir and Verdun avir) and Manilae L495 (L495 and M895) strains at a Multiplicity of Infection (MOI) of 50 bacteria per cell. *E. coli* LPS (1 µg/mL) was used as a positive control for DCs stimulation. **A**: After 24h, cells were immuno-stained for the analysis of the expression of CD80 and MHC-II through flow cytometry and percentage of positive cells are represented in dot plot. **B**: IL-12p40 and IL-10 concentrations were also quantified in cell supernatants by ELISA assays and results are displayed in histograms with dot plot. Data were compared to results with non-stimulated cells (NS). Each dot represents technical triplicates (A) or quadruplicates (B) from 2 independent experiments with n=5 mice. Mean ± SD is also represented. Statistics indicated with lower-case letters allow to compare, for each marker and each cytokine, all conditions within WT (upper panel) and TRIF/*Lps2* (lower panel). Conditions with the same lower-case letters are not significantly different (P > 0.05) using Kruskal-Wallis comparison test followed by post-hoc Fisher's LSD test.

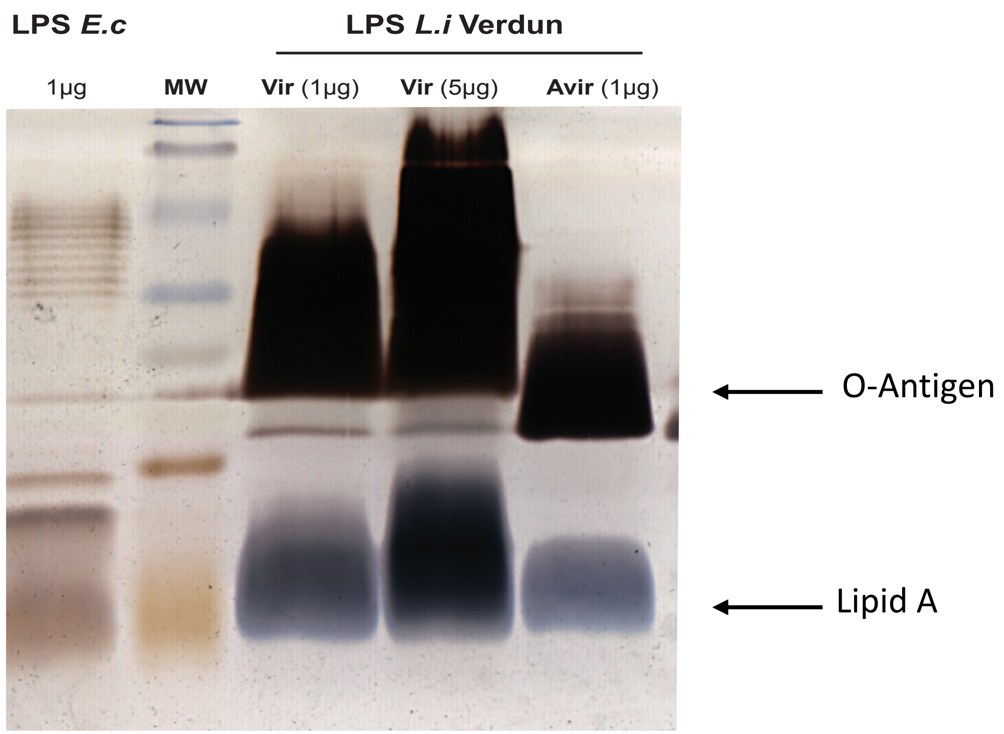

Supplementary Figure 6 : The O antigen part of LPS from the Verdun avirulent strain is shorter than the virulent one
Silver staining of 10% acrylamide gel loaded with LPS from *E. coli*, *L. interrogans* Verdun Vir and Avir, prepared as previously described (Bonhomme *et al.*, PLoS pathogens, 2020).
MW; molecular weight (Rainbow markers, Biorad),
